# Social–ecological predictors of spotted hyena navigation through a shared landscape

**DOI:** 10.1002/ece3.11293

**Published:** 2024-04-25

**Authors:** Christine E. Wilkinson, Wenjing Xu, Amalie Luneng Solli, Justin S. Brashares, Christine Chepkisich, Gerald Osuka, Maggi Kelly

**Affiliations:** ^1^ Department of Environmental Science, Policy, and Management University of California, Berkeley Berkeley California USA; ^2^ California Academy of Sciences San Francisco California USA; ^3^ School of Veterinary Science University of California, Davis Davis California USA; ^4^ Wildlife Research and Training Institute, Lake Nakuru National Park Nakuru Kenya; ^5^ Department of Natural Resources Egerton University Nakuru Kenya

**Keywords:** barrier behavior, carnivore, coexistence, fence ecology, landscape of fear, tolerance

## Abstract

Human–wildlife interactions are increasing in severity due to climate change and proliferating urbanization. Regions where human infrastructure and activity are rapidly densifying or newly appearing constitute novel environments in which wildlife must learn to coexist with people, thereby serving as ideal case studies with which to infer future human–wildlife interactions in shared landscapes. As a widely reviled and behaviorally plastic apex predator, the spotted hyena (*Crocuta crocuta*) is a model species for understanding how large carnivores navigate these human‐caused ‘landscapes of fear’ in a changing world. Using high‐resolution GPS collar data, we applied resource selection functions and step selection functions to assess spotted hyena landscape navigation and fine‐scale movement decisions in relation to social–ecological features in a rapidly developing region comprising two protected areas: Lake Nakuru National Park and Soysambu Conservancy, Kenya. We then used camera trap imagery and Barrier Behavior Analysis (BaBA) to further examine hyena interactions with barriers. Our results show that environmental factors, linear infrastructure, human–carnivore conflict hotspots, and human tolerance were all important predictors for landscape‐scale resource selection by hyenas, while human experience elements were less important for fine‐scale hyena movement decisions. Hyena selection for these characteristics also changed seasonally and across land management types. Camera traps documented an exceptionally high number of individual spotted hyenas (234) approaching the national park fence at 16 sites during the study period, and BaBA results suggested that hyenas perceive protected area boundaries' semi‐permeable electric fences as risky but may cross them out of necessity. Our findings highlight that the ability of carnivores to flexibly respond within human‐caused landscapes of fear may be expressed differently depending on context, scale, and climatic factors. These results also point to the need to incorporate societal factors into multiscale analyses of wildlife movement to effectively plan for human–wildlife coexistence.

## Graphical abstract

Regions where human infrastructure and activity are increasing rapidly or newly appearing constitute novel environments where wildlife must learn to navigate and coexist with people; yet, we do not fully understand how even the most behaviorally flexible species move through these social–ecological landscapes. We used high‐resolution GPS collar data, camera trap imagery, and a barrier behavior analysis (BaBA) to assess the combined influence of ecological, infrastructural, and human tolerance covariates on spotted hyena (*Crocuta crocuta*) movement through a rapidly developing landscape in Nakuru County, Kenya. We found that hyenas appear to perceive fences as risky yet still cross them out of necessity, and that hyenas respond to different social–ecological factors depending on scale and management context, with human tolerance playing an important role in fine‐scale movement decisions.

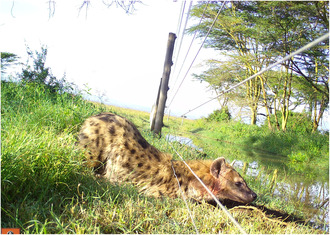

## Muhtasari

Mwingiliano kati ya binadamu na wanyamapori unaongezeka kwa ukali kutokana na mabadiliko ya hali ya hewa na ukuaji wa miji. Maeneo ambapo miundombinu ya binadamu na shughuli zinaongezeka kwa haraka au kutokea kwa mara ya kwanza yanajumuisha mazingira mapya ambayo wanyamapori lazima wajifunze kuishi pamoja na watu, hivyo kutoa masomo bora ya kifani ya jinsi ya kufikiria mwingiliano wa baadaye kati ya binadamu na wanyamapori katika mandhari ya pamoja. Kama mnyama ambaye watu wanaona vibaya na mwenye tabia inayoweza kubadilika, fisi madoa (*Crocuta crocuta*) ni spishi ya mfano katika kuelewa jinsi wanyama pori wanavyopitia ‘mandhari ya hofu’ zilizosababishwa na binadamu katika ulimwengu unaobadilika. Tumetumia data iliyokusanywa na vifaa vya GPS vilivyowekwa kwenye shingo ya fisi, tumetumia vigezo vya uteuzi wa rasilimali na vigezo vya uteuzi wa hatua kuchunguza njia za mandhari za fisi madoa na maamuzi ya harakati za kina kwa kuzingatia vipengele vya kijamii na mazingira katika eneo linaloendelea kwa kasi linalojumuisha maeneo mawili yaliyolindwa: Hifadhi ya Kitaifa ya Ziwa Nakuru na Soysambu Conservancy, Kenya. Kisha tumetumia picha za kamera na Uchambuzi wa Tabia ya Kizuizi (BaBA) kuchunguza zaidi mwingiliano wa fisi madoa na vizuizi. Matokeo yetu yanaonyesha kuwa sababu za mazingira, miundombinu ya linear, maeneo yenye mzozo kati ya binadamu na wanyama pori, na uvumilivu wa binadamu zote zilikuwa ni viashiria muhimu vya uteuzi wa rasilimali wa fisi madoa kwa kiwango cha mandhari, wakati viashiria vya uzoefu wa binadamu vilikuwa na umuhimu mdogo katika harakati za kina za fisi madoa. Uchaguzi wa fisi kwa vipengele hivi pia ulibadilika kulingana na msimu na aina za usimamizi wa ardhi. Kamera zilirekodi idadi kubwa sana ya fisi madoa (234) wanaokaribia uzio wa hifadhi ya kitaifa katika tovuti 16 wakati wa kipindi cha utafiti, na matokeo ya BaBA yalionyesha kuwa fisi wanachukulia uzio wa eneo lililolindwa kama hatari lakini wanaweza kuuvuka kwa lazima. Matokeo yetu yanasisitiza kuwa uwezo wa wanyama pori wa kujibu kwa mazingira ya hofu yaliyosababishwa na binadamu unaweza kujitokeza tofauti kulingana na muktadha, kiwango, na sababu za hali ya hewa. Matokeo haya pia yanasisitiza umuhimu wa kuingiza sababu za kijamii katika uchambuzi wa harakati za mnyama pori kwa kiwango mbalimbali ili kupanga kwa ufanisi kwa ushirikiano kati ya binadamu na wanyamapori.

## 1 INTRODUCTION

Human land uses and development are altering landscapes worldwide, driving rapid urbanization (Ramalho & Hobbs, 2012) and restricting the movements of wide‐ranging species such as large carnivores (Crooks et al., 2011; Ripple et al., 2014). One outcome of the expanding human footprint is that carnivores and people are increasingly overlapping with one another, intensifying the human‐caused *landscape of fear* for carnivores, in which the risk of anthropogenic mortality influences carnivore behaviors and movement (Oriol‐Cotterill et al., 2015; Smith et al., 2017). Fostering landscapes of coexistence for people and carnivores (Oriol‐Cotterill et al., 2015) amidst global development becomes even more difficult as climate change simultaneously exacerbates human–wildlife conflict through numerous social and environmental pathways (Abrahms et al., 2023). Additionally, while there have been various studies on human–carnivore interactions and anthropogenic impacts on carnivores along the wildlife–urban interface (WUI) and within urban areas (e.g., Bateman & Fleming, 2012; Coon et al., 2019; Klees van Bommel et al., 2020; Lute et al., 2020; Smith et al., 2019), little is known about the effects of newly and currently developing urbanization on carnivore movement and propensities for conflict. Yet, regions in which human infrastructure and activity are rapidly densifying or the WUI is newly expanding—here termed *coexistence frontiers*—present carnivores with novel landscapes consisting of novel risks. Assessing how carnivores adaptively traverse *coexistence frontiers* is critical for determining anthropogenic impacts on movement, connectivity, and survival (Sanjayan & Crooks, 2005), while also providing researchers and managers with insight into how to better plan for coexistence amidst global change.

Understanding carnivore landscape navigation within *coexistence frontiers* requires social–ecological approaches—i.e., considering infrastructure, human activity, and other societal and anthropogenic elements—rather than only considering ecological factors (Lute et al., 2020; O'Neal Campbell, 2014). Wildlife sharing space with people must navigate three main elements present on the landscape: ecological factors, human infrastructure, and human tolerance, all of which determine social–ecological landscape permeability (e.g., Ghoddousi et al., 2021; Williamson et al., 2023). In these social–ecological spaces, people interact with, respond to, and alter ecological features and processes that influence carnivore movement at different scales, such as vegetation availability (e.g., Bateman & Fleming, 2012; Suraci et al., 2020) and climatic season (e.g., through changing livestock movements; Schuette et al., 2013). Various tools have also been employed in human‐dominated landscapes to mitigate carnivore interactions with people, including fencing protected areas or other barriers and policies to separate people from wildlife (e.g., McInturff et al., 2020). The permeability of these structures can result in new patterns of wildlife landscape navigation that may have both intended and unintended consequences for movement and coexistence (e.g., Kesch et al., 2013; Lazure & Weladji, 2023).

While certain carnivore species may be able to persist in human‐altered habitats (Athreya et al., 2013; Breck et al., 2019; Chapron et al., 2014; Devens et al., 2019), human intolerance—and resulting human actions toward carnivores—may be a strong enough limiting factor that it can override adaptability for carnivore populations or survival of individuals navigating developing landscapes (Moss et al., 2016). Thus, human perceptions are likely to be an important factor in determining how carnivores navigate landscapes by influencing conservation policies or human‐caused mortality (Behr et al., 2017; Oriol‐Cotterill et al., 2015). On *coexistence frontiers*, people may not have developed tolerance or acceptance for species which they have not encountered before or which they are now encountering more frequently (e.g., Lute & Carter, 2020). In this case, tolerance, as measured by perceptions, attitudes, actions, and desired actions toward wildlife, may serve as an indicator for people's tendencies to conduct illicit persecutory actions toward wildlife (Benson et al., 2023; Ditmer et al., 2022; Manfredo et al., 2021), such as poisoning (see Ogada, 2014) or habitat destruction (see Ripple et al., 2014). Along with risk perceptions and tolerance regarding carnivores, people's perceptions of conflict occurrences and spatial distributions may differ from the frequency and distribution of realized or confirmed conflicts (Wilkinson, Brashares, et al., 2021). This discrepancy can stem from limitations in resources available for verifying conflict occurrences (i.e., within government‐run wildlife management agencies) or be influenced by the tendency of conflict‐related perceptions and chosen actions about particular wildlife species to spread across social networks and be intricately linked with a person's views on wildlife as a whole (Carter et al., 2020; Dickman et al., 2014). Thus, including both confirmed and perceived conflict in movement models can be useful for determining carnivore behavior in relation to prior conflict‐prone locations (Miller, 2015), while perceived conflict may also reflect underlying negative values and, possibly, behaviors toward carnivores.

Contextualizing carnivore behaviors, movement, survival, and interactions with people in human‐caused landscapes of fear also requires consideration of spatiotemporal and ecological scales (Carter & Linnell, 2016). For instance, anthropogenic development may have community‐level effects by pushing some species into limited remaining natural habitats (Parsons et al., 2018), yet at a fine scale, carnivores can exhibit individual‐level adaptations to anthropogenic development or even be synanthropic (i.e., Moss et al., 2016; Nisi et al., 2021; Suraci et al., 2020; Wang et al., 2015). Through these scale‐dependent processes, human‐dominated landscapes near protected areas can result in a source‐sink dynamic, whereby carnivore populations from protected areas that venture into more densely human‐populated regions are more likely to die through anthropogenic causes (Lamb et al., 2020). However, individuals may succeed in these spaces by taking advantage of anthropogenic resources at finer scales, depending on human tolerance (i.e., Moss et al., 2016). Thus, for large carnivores, which are often highly mobile, social–ecological landscape permeability across scales is essential to maintain populations. For example, carnivore species living in both human‐dominated environments and protected areas may avoid anthropogenic features such as roads and fences (e.g., Baker & Leberg, 2018; McInturff et al., 2020; Young et al., 2019) or change their activity patterns to utilize infrastructure for specific purposes or adjust for human presence (Abrahms et al., 2016; Gaynor et al., 2018). Yet carnivores may also either avoid or be attracted to human infrastructure at different scales (Poessel et al., 2014), underpinned by the density of the infrastructure (Morales‐Gonzalez et al., 2020; Xu et al., 2023), the infrastructure's impact on resource availability (i.e., Belton et al., 2016), or individual animal characteristics, such as life stage (Thorsen et al., 2022).

As a widely reviled (Glickman, 1995; Macdonald et al., 2022) and behaviorally plastic apex predator (Holekamp & Dloniak, 2010), spotted hyenas (*Crocuta crocuta*, hereafter “hyenas”) are a model species for understanding the nature of carnivore adaptiveness to human‐caused landscape change and negative human perceptions. Hyenas have an intricate social structure that contributes to notable problem‐solving abilities (Benson‐Amram & Holekamp, 2012; Drea & Carter, 2009) and are generally considered one of the most behaviorally flexible carnivore species (Holekamp & Dloniak, 2010), yet this flexibility has inhibited many generalizable results about their habitat selection. Furthermore, despite our foundational understanding of their behavioral ecology, there have also been few empirical conclusions regarding the extent and mechanisms of their adaptiveness to human activities, infrastructure, and tolerance (Searle et al., 2023; Wilkinson et al., 2023). Green et al. (2018) found that hyena populations in Maasai Mara, Kenya increased in an area of anthropogenic disturbance, possibly linked to increased livestock consumption. In densely populated areas in Ethiopia where native prey is depleted, hyenas have become almost entirely dependent on anthropogenic food (e.g., Yirga et al., 2013). However, other studies have found negative, neutral, or nuanced responses to people. In one study in Kenya, hyena activity shifted in response to livestock grazing and other anthropogenic activities (Kolowski et al., 2007), while a study in South Africa found that hyena propensity to visit anthropogenic sites varied depending on season, age, or individual (Belton et al., 2018). This variation is underpinned by the notable variation in individual personality traits among spotted hyenas—such as aggressiveness, boldness, and sociability—that are often linked to social rank and can dictate survival (Yoshida et al., 2016). Despite these socially‐driven adaptive mechanisms, spotted hyena range is also estimated to have contracted by >20% in less than half a century (Wolf & Ripple, 2017). Determining whether a species is surviving or thriving in the presence of humans is complex, as even highly synanthropic species may be exposed to greater levels of stress, toxicants, and disease while living in anthropogenic landscapes (Murray et al., 2016, 2019). Thus, while movement is solely the broadest mechanism with which to infer carnivore survival and wellbeing within *coexistence frontiers*, understanding how hyenas and other behaviorally plastic, generalist mammalian carnivores navigate anthropogenic landscape change is fundamental to forecasting coexistence and the resilience of ecosystems in rapidly developing landscapes.

We sought to provide insight into spotted hyena abilities to navigate dynamic human‐caused landscapes of fear by examining the following questions in a rapidly developing region encompassing Lake Nakuru National Park and Soysambu Conservancy, Kenya: (1) How do spotted hyena home range sizes and inter‐clan home range overlap vary across seasons and land management types? (2) How do spotted hyenas navigate linear infrastructure (roads and fences), human–carnivore conflicts (verified livestock predation, perceived livestock predation), human tolerance (people's perceptions of risks faced from carnivores), and ecological factors across scales, seasons, and management types (i.e., fully protected vs. multi‐use)? (3) How many individually‐identified spotted hyenas cross through the region's conservation fence, and how does spotted hyena fence navigation manifest at a fine scale? We predicted that (1) linear infrastructure and human experience—rather than just ecological factors—would play significant roles in hyena habitat selection and movement decisions (Green & Holekamp, 2019; Kushata et al., 2018; Naha et al., 2023; Williams et al., 2021; Young et al., 2020), (2) hyena selection for and against landscape characteristics will be scale‐dependent, driven by scale‐dependent heterogeneities in human activities and habitat availability (e.g., Belton et al., 2016; Green & Holekamp, 2019), (3) hyenas would expand their ranges in the rainy season and select more strongly for linear infrastructure and locations of known carnivore‐livestock conflicts in the rainy season than in the dry season (Kolowski & Holekamp, 2006; Mponzi et al., 2014; Naha et al., 2023; Trinkel et al., 2006; Watts & Holekamp, 2009), (4) hyenas with dens in the fully protected national park would be more avoidant of barriers, less avoidant of roads, and more attracted to verified and perceived livestock predation hotspots outside of the park than would hyenas with dens in the multi‐use conservancy (Boydston et al., 2006; Green et al., 2018; Pangle & Holekamp, 2010; Turner et al., 2019), and (5) fence crossing would be limited to a few select individuals due to the perceived riskiness of fence navigation (Belton et al., 2016; Castillo, 2020; Naciri et al., 2023; Naha et al., 2023) (Table 1). We employ resource selection functions (RSFs), step‐selection functions (SSFs), and Barrier Behavior Analysis to determine hyena space use and landscape navigation at different scales (Reinking et al., 2019; Squires et al., 2020). We then use this information to infer whether and how to consider a suite of social and ecological factors when designing for hyena landscape permeability, and present the implications of these inferences for global human–carnivore coexistence.

**TABLE 1:** Hypotheses regarding spotted hyena landscape navigation in the study area.

Question	Hypotheses	Justification
How do spotted hyena home range sizes and inter‐clan home range overlap vary across seasons and land management types?	Home ranges for hyenas denning in Soysambu are smaller than home ranges for hyenas denning in Lake Nakuru National Park. Home ranges are larger during the rainy season. Inter‐clan home ranges overlap more during the rainy season.	Hyenas living in the presence of livestock and herders moved faster, traveled over longer distances, and were more likely to stay within their territories than conspecifics living in areas without livestock and herders.^2^ For spotted hyena home ranges with human infrastructure, the most modified areas were avoided and the least modified areas were preferred.^6^ Hyenas tended to cross out of the protected area during the wet season.^5^ Across many ecosystems, hyenas tend to track migratory prey during the wet season, thus expanding their territories.^19^ Hyenas tested in the low‐disturbance area exhibited more risk‐taking behaviors in relation to a novel, anthropogenic object than did hyenas living in the high‐disturbance area.^23^
How do spotted hyenas navigate linear infrastructure, human–carnivore conflicts, and human tolerance, and what is the importance of these factors for landscape navigation?	Anthropogenic factors such as perceptions and tolerance—rather than solely ecological factors—play significant roles in hyena habitat selection and movement decisions. Fence crossing is limited to a few select individuals of low rank due to the perceived and actual riskiness of fence navigation. Previous conflict locations are correlated with hyena habitat selection.	Availability of roads was key for hyena habitat selection; for example, as a means of moving through bushy habitats.^1^ Hyenas living in the presence of livestock and herders moved faster, traveled over longer distances, and were more likely to stay within their territories than conspecifics living in areas without livestock and herders.^2^ Human perceptions about spotted hyenas were correlated with hyena problem solving abilities and flight initiation distances.^3^ Fence permeability was an important consideration for brown hyena management and should be considered for other carnivores.^4^ Hyenas moved faster and had straighter movement patterns when they were close to fences.^5^ For spotted hyena home ranges with human infrastructure, the most modified areas were avoided and the least modified areas were preferred.^6^ Spotted hyena distribution was negatively impacted by villages and proximity to the border of the reserve, and positively impacted by ecotourism camps and cattle posts.^7^ Hyenas experienced higher roadkill instances near anthropogenic amenities.^8^ Hyenas of low social rank were more likely than hyenas of high social rank to engage in long distance travel events.^2^ To be more accurate, landscape permeability needs to take into account both social and ecological factors.^9,10^ Spotted hyena density was negatively affected by anthropogenic disturbance.^11^ Carnivore‐livestock conflict risk can be predicted by a combination of anthropogenic and ecological covariates.^24,25^
How do spotted hyenas navigate ecological features?	Spotted hyenas will be attracted to locations with water sources, higher Normalized Difference Vegetation Index (NDVI), and lower elevations. Ecological features may not predict selection as strongly as anthropogenic factors.	Spotted hyenas are generalists that can survive across a range of habitats, making their habitat selection difficult to predict.^12^ Spotted hyenas often den near seasonal or permanent watercourses.^12^ Hyenas preferred denser vegetation that can confer protection from humans^13^ and provide safety and shade.^1^ Spotted hyena distribution was likely to be higher closer to rivers.^14^ Underpasses located in areas with higher NDVI and near water sources were more likely to be used by spotted hyenas.^15^
How does spotted hyena landscape navigation differ across scales, seasons, and management types?	Hyena selection for or against landscape characteristics will be scale‐dependent, driven by scale‐dependent heterogeneities in human activities and habitat availability. Hyena home ranges will be larger in the rainy season than in the dry season. Hyenas will select more strongly for anthropogenic infrastructure and locations of known carnivore‐livestock conflicts in the rainy season than in the dry season. Hyenas with dens in the fully protected national park will be more avoidant of barriers, less avoidant of roads, and more attracted to livestock predation hotspots outside of the park than will hyenas with dens in the multi‐use conservancy.	Human activity and infrastructure influenced both fine‐scale and broader scale decision making and habitat selection for spotted hyenas.^6^ Spotted hyenas adjusted their fine‐scale and broader scale movements and activity patterns in response to both immediate/fine‐scale and constant/general herder and livestock presence.^2^ Livestock depredation by hyenas was positively correlated with rainfall,^16–18^ and hyenas spent more time outside of the protected area during months with frequent livestock attacks.^16^ Across many ecosystems, hyenas tend to track migratory prey during the wet season, thus expanding their territories.^19^ Hyenas tended to cross out of the protected area during the wet season.^5^ Hyenas stayed closer to the den when vehicle numbers were high.^20^ Hyenas in more disturbed areas were more vigilant while resting, and they nursed their young closer to bushes.^21^ Hyenas were more likely to be closer to the reserve edge when livestock were present in high numbers.^22^ Hyenas tested in the low‐disturbance area exhibited more risk‐taking behaviors in relation to a novel, anthropogenic object than did hyenas living in the high‐disturbance area.^23^

*Note*: 1. Kushata et al. (2018), 2. Green and Holekamp (2019), 3. Young et al. (2020), 4. Williams et al. (2021), 5. Naha et al. (2023), 6. Belton et al. (2016), 7. Castillo (2020), 8. Naciri et al. (2023), 9. Ghoddousi et al. (2021), 10. Williamson et al. (2023), 11. Searle et al. (2023), 12. Holekamp and Dloniak (2010), 13. Mwampeta et al. (2021), 14. Abade et al. (2014), 15. Lala et al. (2022), 16. Kolowski and Holekamp (2006), 17. Watts and Holekamp (2009), 18. Mponzi et al. (2014), 19. Trinkel et al. (2006), 20. Boydston et al. (2006), 21. Pangle and Holekamp (2010), 22. Green et al. (2018), 23. Turner et al. (2019), 24. Miller (2015), 25. Broekhuis et al. (2017).

## 2 MATERIALS AND METHODS

### 2.1 Study site

Our study was conducted in Nakuru County, in the Rift Valley of southwest Kenya, from June 2018–October 2020. The study area (0°26′ S, 36°1′ E) includes two major wildlife protected areas: Lake Nakuru National Park (LNNP, 188 km^2^), which is one of two fully fenced national parks in Kenya, and Soysambu Conservancy (190 km^2^), which is mostly fenced and functions as both a wildlife conservancy and a livestock ranch with over 10,000 cattle, sheep, and goats. Unlike hyenas denning in LNNP, which is fully protected and a tourist destination, spotted hyenas denning in Soysambu Conservancy are exposed to considerable hazing, multi‐use human activities, and other constant anthropogenic risks, and exhibit marked skittishness. Fences used in both protected areas are typically ~2 m tall and consist of parallel electrified wires, though some stretches of fence are composed of other materials, are in various states of maintenance, or have an additional component of woven wire mesh to reduce wildlife digging. During the study period, various locations along the fence were either non‐electrified or temporarily electrified, allowing many species to dig underneath the fence and successfully enter and exit the protected areas (Wilkinson, McInturff, et al., 2021). The two large alkaline lakes in the region, Lake Nakuru and Lake Elementeita, are designated UNESCO World Heritage sites. The region supports many species of large mammals, including large carnivore species such as African lion (*Panthera leo*), spotted hyena, and leopard (*Panthera pardus*), and several mesocarnivore species, such as serval (*Leptailurus serval*) and black‐backed jackal (*Lupulella mesomelas*). Many carnivore populations in the region remain stable despite heavy historical persecution (Ogutu et al., 2017). The region is arid and is characterized by woodland, savanna, and dense brush habitats, as well as two rainy seasons (approximately March–May, October–December) and two dry seasons (approximately January–February, June–September) each year, with increases in drought events over the past several decades (Ayugi et al., 2020). Additionally, this area is experiencing rapid immigration and is increasingly urbanizing, with Nakuru, just to the north of Lake Nakuru National Park, being designated a city in 2021. The increases in both the spread and concentration of the human population, anthropogenic activities, and human infrastructure are juxtaposing with wildlife resource needs in novel ways for this region, thus producing a *coexistence frontier*.

### 2.2 Field methods and data

#### 2.2.1 Collar deployment and programming

After field assessments, engagement with regional wildlife managers, and discussions with community members in the surrounding region, we determined that there was no evidence of hyena clans denning outside of and nearby the two protected areas. However, existing preliminary data (i.e., from camera traps on protected area boundaries) and accounts (i.e., Kenya Wildlife Service and Soysambu Conservancy personal communication) showed that hyenas denning within the two protected areas routinely exited into the nearby communities. From February–March 2019, 3 female and 4 male spotted hyenas were collared (Savanna Tracking GPS‐GSM FlexTrack), representing 5 clans denning in LNNP and Soysambu Conservancy. Six of these collars (still representing 5 clans) remained in function for most of the study period. We retained data from the two spotted hyenas collared within the same clan since spotted hyenas live in fission–fusion societies (Smith et al., 2008) and their individual social ranks can dictate their space use, landscape navigation, and risk‐taking behaviors (Belton et al., 2018; Boydston et al., 2003; Green et al., 2018). Fixes were taken between 6 pm–7 am, which are the primary active hours of hyenas in this study area. Hourly fix rates were taken from February to April 2019, after which 5‐min fixes were used until May 2020. After this point, the collars were reprogrammed for 1‐h fixes, 24 h per day.

#### 2.2.2 Covariates

Ten environmental, linear infrastructure, and human perception covariates (Table 2) were used in analyses of hyena landscape use and navigation. Environmental covariates included normalized difference vegetation index (NDVI; 30 m, Landsat 8 Surface Reflectance Tier 1, rainy and dry seasonal averages for 2019), slope (30 m, Shuttle Radar Topography Mission [STRM]), elevation (30 m, STRM), distance to rivers, and distance to lakes. NDVI in this study site can serve as a proxy for land cover, because areas of higher NDVI are generally brush or forest, whereas lower NDVI areas are typically grasslands. Linear infrastructure covariates included distance to boundaries and distance to roads. Human perception/experience covariates included distance to verified livestock predation locations, distance to regions people perceive as being risky due to hyenas (whether or not any risky events occurred), and distance to participatory mapped livestock predation locations (i.e., perceived livestock predation) during the study period. The latter two variables were derived in 2018–2019 using participatory mapping data from 378 community members living within 2 km of the protected area boundaries, while the verified predation dataset (2008–2018) was provided by the local wildlife authority, Kenya Wildlife Service, who conducts field verifications of predation reports (Wilkinson, Brashares, et al., 2021). All three conflict‐ and risk‐related datasets were initially collected as vector datasets and comprised areas solely outside, but adjacent to the boundaries of, the two protected areas.

**TABLE 2:** Covariates analyzed for resource selection and step selection functions. All rasters were resampled to 30 m^2^ spatial resolution.

Covariate type	Covariate	Source
Ecological	Normalized difference vegetation index (NDVI); rainy season average for 2019	Landsat 8 Surface Reflectance Tier 1
	NDVI; dry season average for 2019	Landsat 8 Surface Reflectance Tier 1
	Slope	Shuttle Radar Topography Mission
	Elevation	Shuttle Radar Topography Mission
	Distance to rivers	Derived from authors' georeferenced, ground‐verified data
	Distance to lakes	Derived from authors' georeferenced, ground‐verified data
Linear infrastructure	Distance to roads	Open Street Maps and authors' ground‐verified data
	Distance to boundaries	Derived from Kenya Wildlife Service and authors' ground‐verified data
Human experience	Distance to verified livestock predation locations	Derived from Kenya Wildlife Service (see Wilkinson, Brashares, et al., 2021)
	Distance to participatory mapped livestock predation locations	Derived from participatory mapped data (see Wilkinson, Brashares, et al., 2021)
	Distance to regions perceived as risky due to hyenas	Derived from participatory mapped data (see Wilkinson, Brashares, et al., 2021)

The verified predation dataset indicates verified propensity for human–carnivore conflicts in specific locations, which exhibits marked spatial differences from perceived human–carnivore conflicts in this region (Wilkinson, Brashares, et al., 2021). Thus, we included the verified predation dataset to determine whether spotted hyenas select for locations with prior carnivore conflict, i.e., due to anthropogenic or ecological attractants (Broekhuis et al., 2017). Because the killing of or retaliation against wildlife is illegal in Kenya, yet occurs nonetheless, participatory mapped livestock predation and participatory mapped risks from spotted hyenas can serve as ascertainable proxies for spatially explicit intolerance of spotted hyenas by local communities and are associated with the potential for deterrents and aversive behaviors toward hyenas. Notably, these two perception datasets provide slightly different information that is not strongly correlated (*r* = .52): perceived livestock predation consists of occurrences of livestock predation that have been unverified by the authorities, and perceived risk consists of locations that community members feel are risky to go due to hyenas and other carnivores. Thus, the former may also indicate locations that are frequented by the community's livestock, while the latter is oriented solely around people's perceived risk, regardless of whether those risks are related to livestock predation. People may perceive that certain areas are risky because they consist of hyena habitat or are known by the community to be frequented by wildlife (Read et al., 2021; Wilkinson, Brashares, et al., 2021). Since we sought to understand community perceptions regarding threats to their property, we did not include Soysambu Conservancy in the participatory mapped dataset despite its function as a cattle ranch. This is because community members who herd the cattle on Soysambu Conservancy do not own the cattle; rather, they are employed by the landowner. Euclidean distance was used for all distance layers, and road layers were derived through Open Street Maps and by hand tracing. Fences assessed in barrier analyses were mapped in person by driving and walking the boundaries of the protected areas. All distance covariates were created as rasters with 30 m spatial resolution to match the NDVI, slope, and elevation rasters.

## 3 ANALYSES

### 3.1 Assessing space use

To determine the individual home ranges and core ranges of the seven collared hyenas, we used the ‘adehabitatHR’ kernel density estimator with the reference bandwidth (Calenge, 2006) as the smoothing factor in R v.4.1.3 (R Core Team, 2022) to assess 50% (core) and 95% kernel utilization distributions (KUD) to calculate home range area for all individuals and also looked at differences in home range area across seasons and land management type. We calculated overlap among home ranges for all pairs overall and across seasons using the ‘amt’ package (Signer et al., 2019) via two metrics: (1) proportion of overlap and (2) the Bhattacharyya coefficient, which is a measure of overlap between probability distributions (Bhattacharyya, 1943).

### 3.2 Assessing landscape‐scale selection

To determine landscape feature selection at the home range level by spotted hyenas, we derived resource selection functions (RSFs) using the ‘lme4’ package in R, including only the six collared hyenas (3 male and 3 female) whose collars remained active throughout the study (the seventh collar failed within the first month and provided an inadequate sample size for movement analyses). The number of fixes per individual ranged from 57,890 to 61,262, for a total of 342,048 fixes. To reduce autocorrelation and necessary computing power while still gaining a full picture of hyena resource selection, we rarified data to 2‐hour fixes for a total of 16,245 fixes, and used each hyena's 95% KUD to generate availability samples. After assessing coefficient convergence and model deviance across several levels of availability samples (1:1, 2:1, 3:1, 4:1) (Appendix S1), following recommendations by Northrup et al. (2013), we generated random points equal to the number of GPS points used (also see Dellinger et al., 2013). We used the *vif* function in the ‘car’ package (Fox et al., 2007) and the *cor* function to test for multicollinearity and found no evidence of strong collinearity among our covariates. We assessed resource selection using generalized linear mixed‐effects models with a logit link. Using the raster package and base R, we centered and scaled (mean = 0, SD = 1) our covariates for use in the RSFs to facilitate model convergence and interpretability. We included individual as a random effect to control for individual variation in behaviors (Gillies et al., 2006). We assessed a global model (for all hyenas) and compared global seasonal (rainy and dry) models, as well as models for hyenas whose dens were in LNNP (*n* = 3), and for hyenas whose dens were in Soysambu Conservancy (*n* = 3), with our rationale detailed in Table 1. Models were ranked based on their Akaike Information Criterion (AIC; Burnham & Anderson, 2002), and models within Δ AIC ≤2 were retained to use when assessing coefficient values. We tested the accuracy of the global model using our training (80%) and testing data (20%) (Boyce et al., 2002). We then assessed and compared the performance of themed models (using AIC) with the following combinations of variables: ecological variables only (termed “ecological”), ecological and linear infrastructure variables (“physical”), linear infrastructure only (“infrastructure”), infrastructure and human perception/experience variables (“anthropogenic”), and human perception/experience variables only (“human experience”). Finally, we assessed relative importance of each variable using the global model (after Ewald et al., 2014).

### 3.3 Assessing fine scale movement decisions

To understand how hyenas select for and move in relation to landscape features at the fine/step scale, we derived step selection functions (SSFs) using the ‘amt’ package (Fieberg et al., 2021; Signer et al., 2019) and ‘survival’ package (Therneau, 2015) in R (R Core Team, 2022). We prepared the hyena data by creating tracks from the data using the *mk_track* function, thinned the data to 15‐min fixes for a total of 106,745 steps (mean step length = 340.8 m), and filtered to assure bursts would have a minimum of 3 points (Signer et al., 2019). We chose 15‐min fix rates to accurately capture the fine‐scale movement patterns of our focal individuals, especially in relation to linear infrastructure. Five random steps were generated for each step used, using the *random_steps* function, which draws step lengths from a gamma distribution fitted to the entire dataset and draws turn angle from a von Mises distribution (Thurfjell et al., 2014). With very large sample sizes, as with this dataset, few random steps (or even one random step) can be sufficient for assessing step selection (Thurfjell et al., 2014). Scaled covariates and model comparisons reflected those conducted for the RSF analyses. We first tested a global model to determine the lag at which autocorrelation was no longer observed using an autocorrelation function (Appendix S1), and employed destructive sampling to address autocorrelation (Prima et al., 2017; Whittington et al., 2022). Coupled with reporting on robust standard errors, this method allowed us to reduce autocorrelation without compromising our ability to assess fine‐scale movement patterns (Nisi et al., 2021; Prima et al., 2017; Roever et al., 2010; Suraci et al., 2019). All reported models were run on the destructively sampled data, which contained 8 clusters per individual for a total of 48 clusters, well over the 20–30 minimum clusters recommended by Prima et al. (2017). For destructive sampling, 5 h were removed between each cluster for each individual due to 5 h being the point in time at which autocorrelation was found to decay. We estimated coefficients by fitting conditional logistic regressions on the covariates while also considering the following interaction terms with boundaries and roads: log of step length (i.e., speed of movement) and cosine of the turning angle (i.e., directionality of movement). We included these interaction terms to address differences in hyena movement around linear infrastructure, since carnivores have been shown to change behavioral states around linear infrastructure (e.g., Abrahms et al., 2016; Thorsen et al., 2022). We used quasi‐likelihood independence model criterion (QIC) to rank models and determine top models. We also determined the performance of each of our themed models (i.e., ecological, physical, infrastructure, anthropogenic, and perception models) using QIC, and assessed relative importance of each variable using the global model (Ewald et al., 2014).

### 3.4 Barrier interactions

To understand hyena behavior around fences (including fence crossings of collared hyenas) and determine the locations of weak or robust fences on the protected area boundaries, we used the Barrier Behavior Analysis (BaBA) methodology developed by Xu et al. (2020), employing our 5‐min fix rate dataset. BaBA examines whether, where, and how often animal movements were altered by linear barriers such as fences by classifying movement trajectories within set buffers around target barriers into normal movement (*quick cross*, *average movement*), altered movement (*bounce*, *trace*, or *back and forth*), and trapped movement. *Bounce* is a behavior in which hyenas that encounter the fence immediately move away from the fence, *trace* is a behavior in which hyenas move along the fence in a single direction, and *back and forth* is a behavior in which hyenas stay close to the fence, moving in alternating directions alongside it. To assess the appropriate sensitivity for the BaBA results, we used BaBA with 50, 100, and 150 m fence buffer distances within which GPS locations were classified as fence encounters (Xu et al., 2020). Testing different buffer distances allows us to determine the optimal buffer distance at which to accurately capture *quick cross* events rather than misclassifying them (Xu et al., 2020).

Finally, because our collared individuals likely could not capture the broader boundary‐related behaviors of regional hyena clans (with >250 individual hyenas documented across seven known clans in the two protected areas), we sought to supplement our understanding of hyena interactions with the boundary fences by determining the number of unique individual hyenas approaching fence lines, and their respective frequencies of approach. We used images of spotted hyenas from camera traps placed on 16 of the 19 total documented wildlife crossing points at the LNNP fence from June 2018–November 2019 (Wilkinson, McInturff, et al., 2021). The 16 camera sites were chosen (out of the 19 total wildlife crossing sites) because of their locations within the ranges of the GPS‐collared hyenas and the higher overall presence of spotted hyenas seen on camera at these fence crossing points (Wilkinson, McInturff, et al., 2021). Spotted hyenas in camera trap images were manually individually identified (including verification by at least one other observer) using spot patterns and compared to individuals previously listed in both the LNNP and Soysambu Conservancy hyena ID books (see Appendix S1). Hyenas appearing at the fence were first compared to the clan with a range closest to the camera trap site but then expanded to all others in the book if not identified. The hyena was labeled as unknown if we could not definitively identify the individual. These unknown individuals were later added to the Lake Nakuru or Soysambu Conservancy ID Books under a new ID code and used for further identification of images. We assessed the frequency of fence approaches at each site and by specific individuals, and the number of different fence crossing sites visited by each individual. We also calculated the ratio of persistence, or the number of individual hyenas visiting a site divided by the total number of hyena photographs from that site, with lower values indicating more persistence exhibited at that site.

## 4 RESULTS

### 4.1 Landscape‐scale space use

**FIGURE 1:**
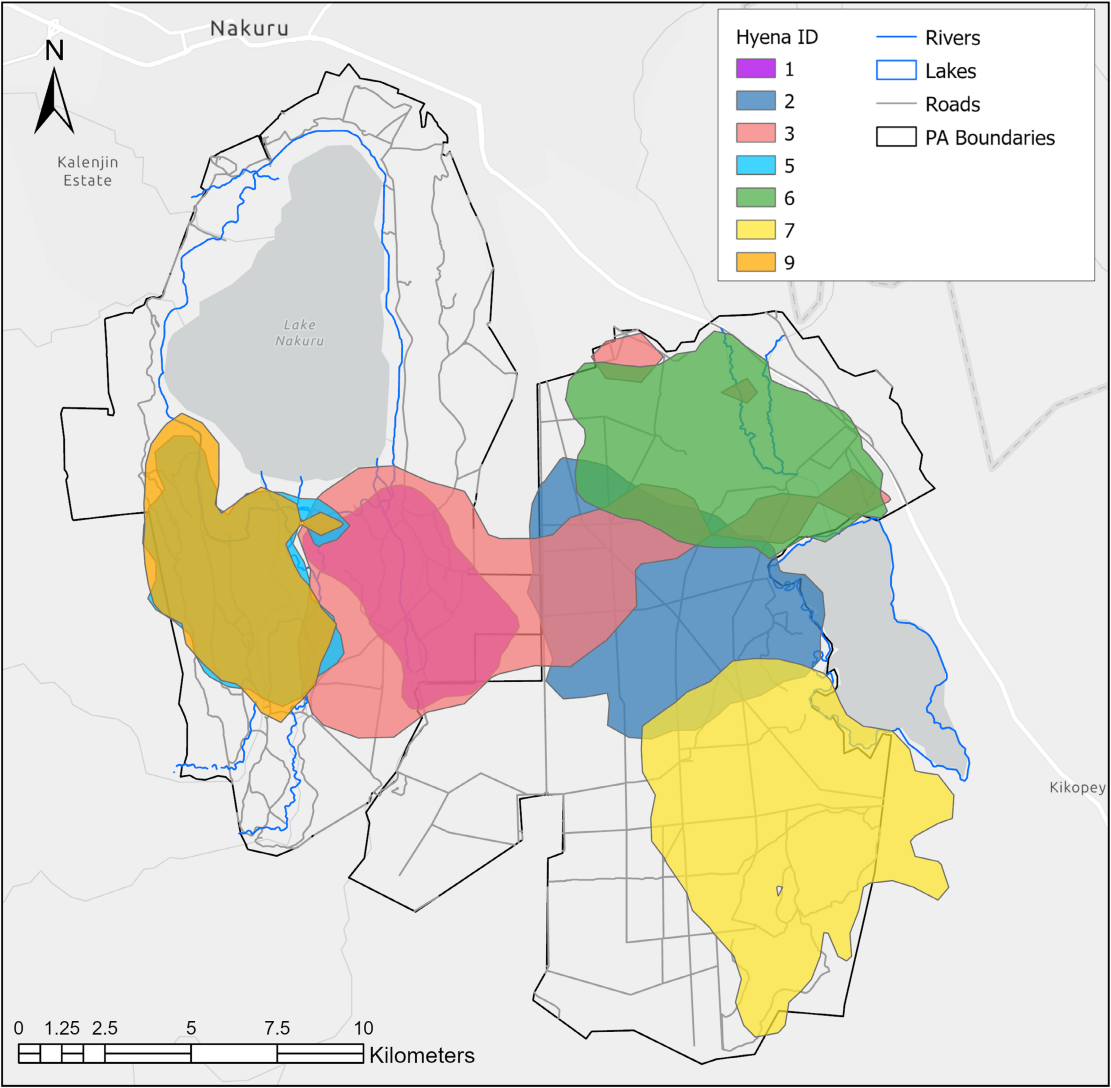
95% kernel utilization distribution home ranges derived from GPS collar data for 7 spotted hyenas representing 5 clans in Lake Nakuru National Park (western side of map) and Soysambu Conservancy (eastern side of map), Kenya. Hyenas 1 and 3 are in the same clan and hyenas 5 and 9 are in the same clan.

Spotted hyena 50% and 95% home ranges (Figure 1) comprised between 5.01–20.97 km^2^ ($\bar{x}$ = 9.69) and 32.57–140.89 km^2^ ($\bar{x}$ = 63.8), respectively. Rainy season 50% and 95% home ranges comprised 5.51–20.92 km^2^ ($\bar{x}$ = 10.31) and 29.63–117.68 km^2^ ($\bar{x}$ = 60.30), respectively. Dry season 50% and 95% home ranges comprised 4.84–21.04 km^2^ ($\bar{x}$ = 9.62) and 31.42–148.98 km^2^ ($\bar{x}$ = 63.08), respectively. The maximum proportion of 95% inter‐clan home range overlap for individual hyenas (assessed pairwise, directionally) was 0.278 ($\bar{x}$ = 0.076, median = 0.043), with a maximum Bhattacharyya coefficient of 0–0.159 ($\bar{x}$ = 0.04, median = 0.02). During the rainy season, the maximum proportion of 95% inter‐clan home range overlap was 0.318 ($\bar{x}$ = 0.056, median = 0.003) with a maximum Bhattacharyya coefficient of 0.157 ($\bar{x}$ = 0.032, median = 0.002). During the dry season, the maximum proportion of 95% inter‐clan home range overlap was 0.702 ($\bar{x}$ = 0.097, median = 0.061) with a maximum Bhattacharyya coefficient of 0.178 ($\bar{x}$ = 0.053, median = 0.076).

### 4.2 Landscape‐scale selection

For all spatial analyses, all variables were retained after testing for pairwise correlation (maximum correlation was 0.52, with all except four pairwise correlations being <0.3). The best performing overall model contained all variables except slope, while the global model was within Δ AIC ≤2 of the best performing model (Table 3). For the RSFs, the most influencing ecological variable was distance to lakes (relative importance = 0.12), the most influencing linear infrastructure variable was distance to boundaries (0.1), and the most influencing human experience variable was distance to perceived livestock predation (0.19), with human experience variables having the highest combined relative importance, followed by ecological variables (Appendix S1). The global model including all covariates for the RSFs (accuracy = 0.57), revealed selection for higher NDVI, roads, lakes, perceived livestock predation, and areas of participatory mapped risk from hyenas, and against elevation, steep slopes, rivers, boundaries, and verified livestock predation (Table 4). Of these, selection for distance to verified livestock predation (i.e., greater distances away from these regions; *β* = 0.305, *p* < .001) and selection against distance to participatory mapped risk (i.e., closer distances to these regions; *β* = −0.277, *p* < .001) showed the strongest effects. When comparing thematic models, the model that best predicted the data was the model containing only ecological and infrastructure covariates (i.e., the “physical” model), followed by the human experience model.

**TABLE 3:** Model selection table for global, dry season only, rainy season only, Lake Nakuru National Park only, and Soysambu Conservancy only resource selection functions.

	Model	df	loglik	AIC	Delta	Weight
RSF	Used ~ NDVI + elev. + rivers + roads + lakes + bounds + vc + pc + risk + (1|ID)	11	−10,976.37	21,974.8	0	0.703
RSF	Used ~ NDVI + elev. + slope + rivers + roads + lakes + bounds + vc + pc + risk + (1|ID)	12	−10,976.24	21,976.5	1.73	0.296
RSF dry	Used ~ NDVI + rivers + roads + lakes + bounds + vc + pc + risk + (1|ID)	10	−11,135.99	22,292	0	0.464
RSF dry	Used ~ NDVI + elev. + rivers + roads + lakes + bounds + vc + pc + risk + (1|ID)	11	−11,135.9	22,293.8	1.82	0.187
RSF dry	Used ~ NDVI + slope. + rivers + roads + lakes + bounds + vc + pc + risk + (1|ID)	11	−11,135.93	22,293.9	1.87	0.182
RSF rainy	Used ~ NDVI + rivers + roads + lakes + bounds + vc + pc + risk + (1|ID)	10	−11,135.99	22,292	0	0.464
RSF rainy	Used ~ NDVI + elev. + rivers + roads + lakes + bounds + vc + pc + risk + (1|ID)	11	−11,135.9	22,293.8	1.82	0.187
RSF rainy	Used ~ NDVI + slope + rivers + roads + lakes + bounds + vc + pc + risk + (1|ID)	11	−11,135.93	22,293.9	1.87	0.182
RSF LNNP	Used ~ NDVI + elev. + slope + rivers + lakes + bounds + vc + pc + risk + (1|ID)	11	−10,715.43	21,452.9	0	0.374
RSF LNNP	Used ~ NDVI + elev. + slope + lakes + bounds + vc + pc + risk + (1|ID)	10	−10,716.61	21,453.2	0.36	0.312
RSF LNNP	Used ~ NDVI + elev. + slope + rivers + roads + lakes + bounds + vc + pc + risk + (1|ID)	12	−10,715.23	21,454.5	1.6	0.168
RSF LNNP	Used ~ NDVI + elev. + slope + roads + lakes + bounds + vc + pc + risk + (1|ID)	11	−10,716.39	21,454.8	1.92	0.143
RSF Soysambu	Used ~ NDVI + elev. + slope + rivers + roads + lakes + bounds + pc + risk + (1|ID)	11	−11,074.48	22,171	0	0.538
RSF Soysambu	Used ~ NDVI + elev. + slope + rivers + roads + lakes + bounds + vc + pc + risk + (1|ID)	12	−11,074.3	22,172.6	1.63	0.238

*Note*: Only models within AIC = 2 are included for each model category. VC = verified conflict, PC = participatory mapped perceived conflict, risk = participatory mapped perceived risk.

**TABLE 4:** Results from global resource selection function model (generalized linear model with a logit link), with individual as a random intercept, for spotted hyenas collared in Lake Nakuru National Park and Soysambu Conservancy, Kenya.

Variable	Coeff	SE	*z*‐Value	*p*‐Value
NDVI	0.15	0.014	9.452	<.001
Elevation	−0.126	0.017	−7.55	<.001
Slope	−0.0002	0.012	0.309	.757
Distance to rivers	0.089	0.028	3.163	.002
Distance to roads	−0.132	0.013	−10.501	<.001
Distance to lakes	−0.231	0.015	−15.654	<.001
Distance to boundaries	0.188	0.016	11.707	<.001
Distance to perceived livestock predation locations	0.318	0.026	12.334	<.001
Distance to verified livestock predation locations	−0.283	0.018	−15.847	<.001
Distance to locations perceived as risky due to hyenas	−0.28	0.02	−13.951	<.001

**FIGURE 2:**
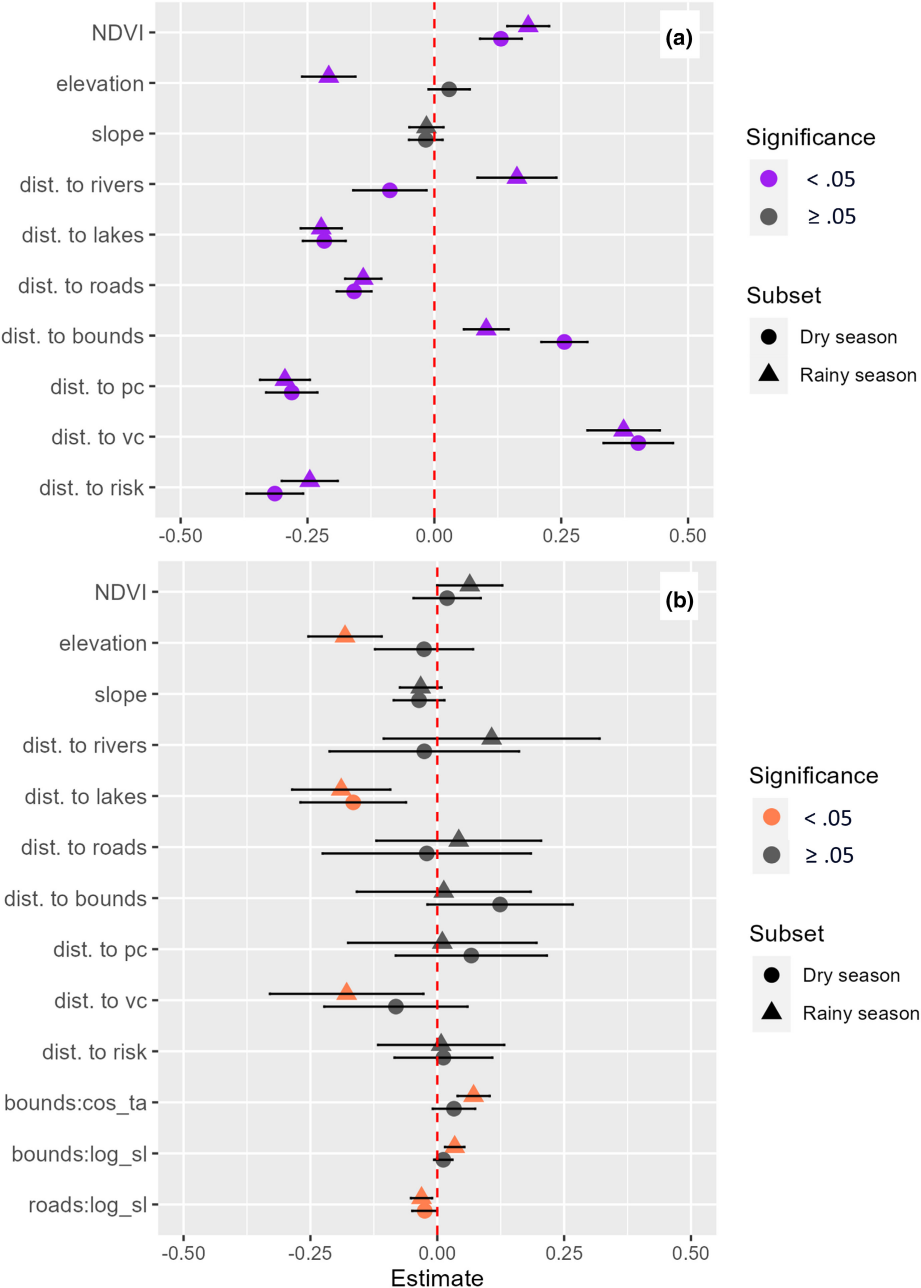
Global (a) resource selection function and (b) step selection function model outputs across rainy and dry seasons for spotted hyena, showing estimates and 95% confidence intervals. Robust standard errors are used for step selection model outputs. Bounds = fenced protected area boundaries, vc = verified livestock predation, pc = participatory mapped perceived livestock predation, risk = participatory mapped risk from spotted hyenas, cos_ta = cosine of the turn angle, log_sl = log of step length.

Seasonal RSFs comparing all covariates across the rainy and dry seasons showed that when compared to the rainy season, during the dry season hyenas exhibit stronger landscape‐scale selection for roads and areas of participatory mapped risk, and a decrease in selection for NDVI, higher elevations, and lakes (Figure 2a). Notably, hyenas exhibited selection against boundaries during both seasons, though boundaries were selected against more strongly in the dry season. The only significant instance of opposite selection was for rivers; hyenas were more likely to select for rivers (i.e., negative relationship with distance to rivers) in the dry season (*β* = −0.092, *p* = .02), and against rivers in the rainy season (*β* = 0.163, *p* < .001). The best performing dry season and rainy season models both excluded elevation and slope (Table 3).

**FIGURE 3:**
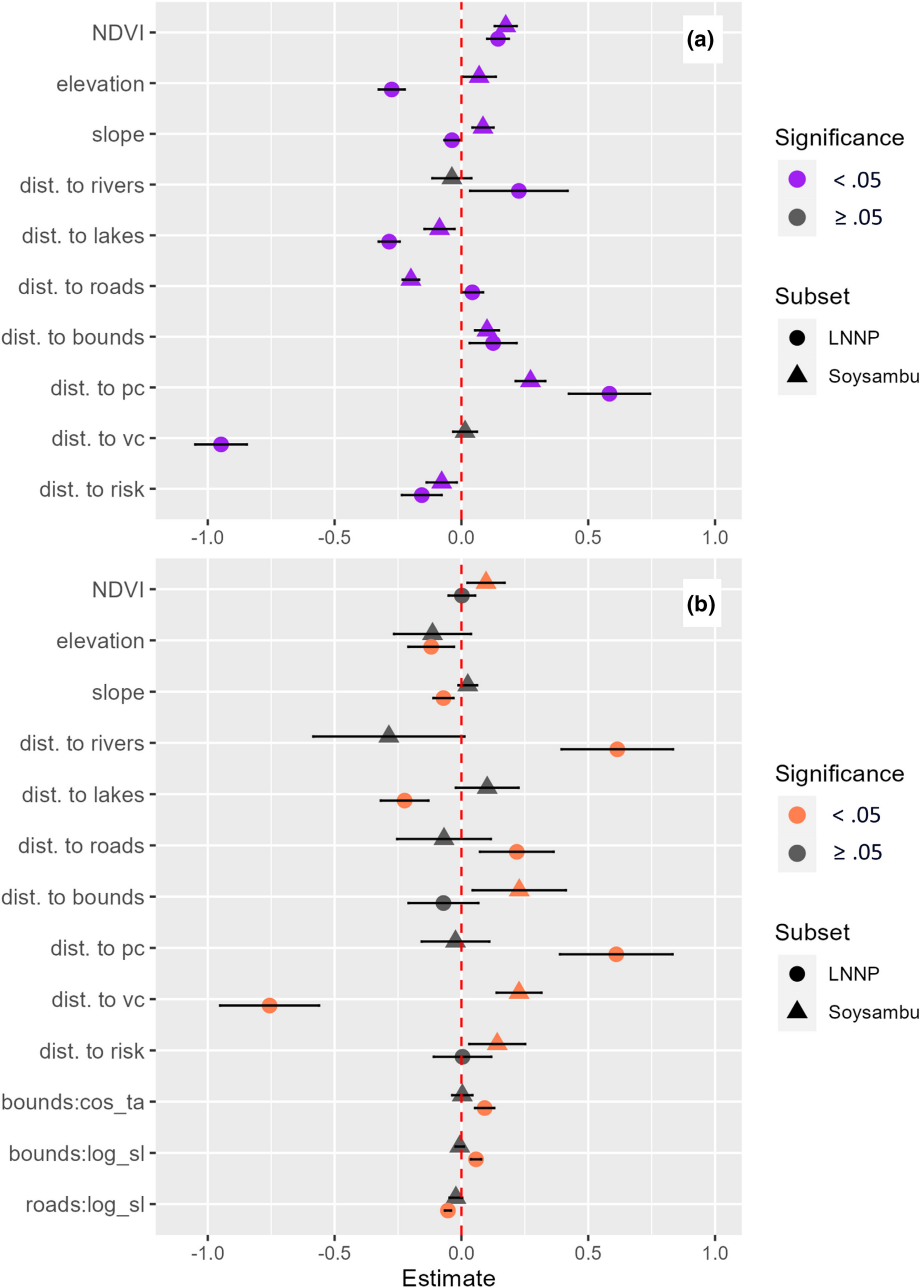
Global (a) resource selection and (b) step selection function model outputs across land management types (LNNP: fully protected, or Soysambu: multi‐use) for spotted hyena, showing estimates and 95% confidence intervals. Robust standard errors are used for step selection model outputs. Bounds = fenced protected area boundaries, vc = verified livestock predation, pc = participatory mapped perceived livestock predation, risk = participatory mapped risk from spotted hyenas, cos_ta = cosine of the turn angle, log_sl = log of step length.

When comparing global models across land management types, hyenas with dens in LNNP showed stronger selection for or against human experience covariates than hyenas with dens in Soysambu (Figure 3a). Specifically, LNNP hyenas exhibited selection for areas of verified conflict (*β* = −1.003, *p* < .001) and participatory mapped risk (*β* = −0.175, *p* < .001), and against areas of perceived conflict (*β* = 0.521, *p* < .001). Meanwhile, Soysambu hyenas exhibited selection for participatory mapped risk (*β* = −0.104, *p* < .001) and against areas of perceived conflict (*β* = 0.314, *p* < .001), with no significant effect for areas of verified conflict. LNNP hyenas also showed statistically significant selection against boundaries at the landscape scale (*β* = 0.179, *p* < .001), which was not exhibited as strongly by Soysambu hyenas (*β* = 0.068, *p* < .001). While Soysambu hyenas selected for higher elevations, steeper slopes, and rivers, LNNP hyenas selected for the opposite. Additionally, LNNP hyenas selected against rivers (*β* = 0.621, *p* < .001), lakes were more strongly selected for (i.e., selecting against distance to lakes) by LNNP hyenas (*β* = −0.298, *p* < .001) than for Soysambu hyenas (*β* = −0.123, *p* < .001), and roads were only significantly selected for by Soysambu hyenas (*β* = −0.209, *p* < .001). The best performing LNNP model excluded distance to roads, while the best performing Soysambu model excluded distance to verified conflict locations (Table 3).

### 4.3 Fine‐scale movement decisions

Initial model performance assessments for SSFs (i.e., QIC) revealed that the best performing models included three interaction terms: boundaries: log(step length), roads: log(step length), and boundaries: cosine(turn angle). Thus, we retained those interaction terms for our assessment of the global model. Additionally, for SSFs the most influencing ecological variable was distance to lakes (relative importance = 0.185), the most influencing linear infrastructure covariate was distance to boundaries (0.195), and the most influencing human experience covariate was distance to verified livestock predation (0.13), with ecological variables having the highest combined relative importance, followed by linear infrastructure (Appendix S1). The global model showed significant selection toward lakes (*β* = −0.178, *p* < .001), lower elevation (*β* = 0.097, *p* = .008), and verified livestock predation locations (*β* = −0.126, *p* = .037). There were also significant positive interactions between distance to boundary and turning angle (*β* = 0.05, *p* = .001) and distance to boundary and step length (*β* = 0.022, *p* = .004), and a significant negative interaction between distance to road and step length (*β* = −0.028, *p* = .005) (Table 5). When comparing themed models, the best performing model was the ecological model containing all three boundary and road movement parameter interaction terms.

**TABLE 5:** Results from global step selection function model (using conditional logistic regressifor spotted hyenas collared in Lake Nakuru National Park and Soysambu Conservancy, Kenya).

Variable	Coeff	SE	Robust SE	*z*‐Value	*p*‐Value
NDVI	0.041	0.006	.024	1.706	.088
Elevation	−0.097	0.012	0.036	−2.672	.008
Slope	−0.034	0.005	0.017	−1.997	.046
Distance to rivers	0.037	0.035	0.082	0.451	.652
Distance to roads	0.013	0.011	0.081	0.154	.877
Distance to lakes	−0.178	0.02	0.041	−4.337	<.001
Distance to boundaries	0.074	0.016	0.065	1.139	.255
Distance to perceived livestock predation locations	0.042	0.023	0.068	0.615	.539
Distance to verified livestock predation locations	−0.126	0.022	0.06	−2.082	.037
Distance to locations perceived as risky due to hyenas	0.01	0.015	0.045	0.213	.831
Distance to boundaries:log_sl	0.022	0.002	0.007	2.849	.004
Distance to boundaries:cos_ta	0.05	0.005	0.016	3.19	.001
Distance to roads:log_sl	−0.028	0.002	0.01	−2.795	.005

Seasonal SSFs comparing all covariates across the rainy and dry seasons showed that in the rainy season hyenas exhibit an increase in strength of selection for lakes and against high elevations and roads, a significant selection toward regions of verified livestock predation (*β* = −0.179, *p* = .021), and an increase in the magnitude and significance of all three assessed interactions between linear infrastructure and movement parameters when compared to both the dry season model and the global model (Figure 2b).

When comparing global models across land management types, LNNP hyenas differed from Soysambu hyenas in that they tended to move toward lakes (*β* = −0.224, *p* < .001) and verified conflict (*β* = −0.756, *p* < .001), and away from roads (*β* = 0.218, *p* = .003), steeper slopes (*β* = −0.071, *p* < .001), and areas of perceived conflict (*β* = 0.61, *p* < .001). In contrast, Soysambu hyenas showed a strong selection away from areas of verified livestock predation (*β* = 0.198, *p* = .001), and selected significantly for NDVI (*β* = 0.097, *p* = .01) and against boundaries (*β* = 0.228, *p* = .02), while LNNP hyenas did not (Figure 3b). Additionally, LNNP hyenas exhibited the most pronounced magnitude of interaction for the assessed interaction covariates and movement parameters, while these interactions showed no significance for Soysambu hyenas. A case study on a single hyena that was known to frequently cross between the two protected areas (Appendix S1) showed an increase in speed of movement (log of step length) when nearer to roads (*β* = −0.142, *p* < .001) and increase in turning angle when farther from boundaries (*β* = 0.035, *p* < .001) during the dry season as compared to the rainy season.

### 4.4 Barrier interactions

**FIGURE 4:**
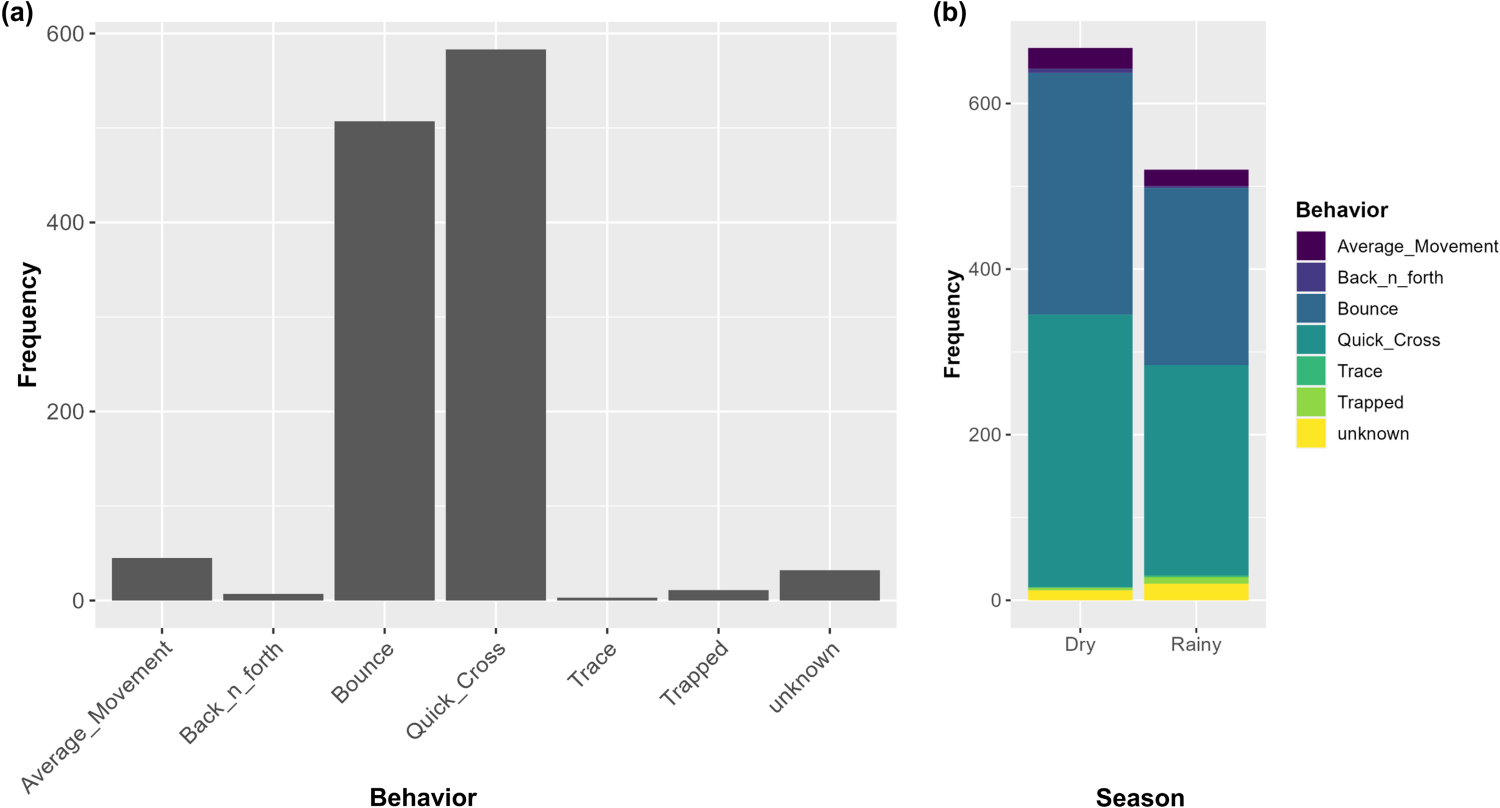
(a) Total frequency of fence behaviors revealed through Barrier Behavior Analysis (BaBA) using a buffer distance of 50, and (b) seasonal frequency of fence behaviors for spotted hyenas living in and near Lake Nakuru National Park and Soysambu Conservancy, Kenya.

All six focal individuals approached the protected area boundary fences. For the Barrier Behavior Analysis, a 50‐m fence buffer best captured *quick cross* events (i.e., larger buffer sizes gave an inaccurately low estimation of *quick cross* events; Xu et al., 2020), or events where hyenas quickly crossed a fence after approaching it. Spotted hyena individuals encountered fences on average 193 (*σ* = 168.5) times during the study period. Overwhelmingly, hyenas encountering fences either exhibited *quick cross* (*n* = 583, or 49% of all fence encounters) or *bounce* (*n* = 507, 42.7%), with *average movement* (*n* = 45, 3.8%), *trace* (3 times, 0.25%), and *back and forth* (*n* = 7, 0.59%) exhibited occasionally (Figure 4). There was marked individual variation in overall fence encounters, as illustrated by high standard deviations in average fence encounter frequency. Mann–Whitney *U* tests demonstrated that *bounce* and *quick cross* behaviors showed no significant difference across seasons. Judging by differences in movement in relation to different fence segments, some segments may be more permeable than others. The highest concentration of quick cross behaviors appeared to be on the fence lines between the two protected areas, indicating high permeability for those fence segments. Meanwhile, the bounce behaviors had a considerably wider spread along the boundaries (see Appendix S1), indicating regions where hyenas may have attempted to cross but could not due to fence impermeability. Notably, fence behaviors revealed several crossing points connecting LNNP to Soysambu Conservancy.

Camera trap data revealed 234 individual hyenas (out of >250 documented hyenas) spanning at least 4 clans (out of 7 documented clans)—and, inherently, various social ranks (Holekamp et al., 2007)—approaching the fence across the 16 studied fence crossing sites, with one site having a minimum of 66 different individuals appearing at the fence (Table 6; Appendix S1). Across all fence crossing sites, 63 individuals (26.9% of all hyenas observed at the fence) appeared at the fence in more than 10 images during the study period. The number of individual hyenas appearing at a site ranged from 1 to 66 ($\bar{x}$ = 20), and the ratio of persistence ranged from 0.016 to 0.5 ($\bar{x}$ = 0.18) (Table 6).

**TABLE 6:** The number of spotted hyena individuals appearing on camera, the frequency of hyena photographs, the % of total camera trapped individuals represented, and the ratio of persistence (i.e., the # of individuals divided by the frequency of hyena photographs) at the Lake Nakuru National Park fence line across 16 sites.

Site	# Individuals	Frequency of hyena photographs	% of total camera trapped individuals represented^a^	Ratio of persistence
C1	24	622	10.3	0.039
C2	56	732	23.9	0.076
C3	37	730	15.8	0.051
C4	22	325	9.4	0.068
C6	13	152	5.6	0.086
C7	1	2	0.04	0.5
C8	60	769	25.6	0.078
C9	2	27	0.09	0.074
C10	1	3	0.04	0.33
C12	1	4	0.04	0.25
C13	13	82	5.6	0.16
C14	1	2	0.04	0.5
C15	7	30	3.0	0.23
C16	14	42	6.0	0.33
C17	66	4101	28.2	0.016
CX	8	48	3.4	0.17
Total # Individuals on Camera^a^	234			

*Note*: Lower values of the ratio of persistence indicate a more persistent sample of individuals appearing at the camera site.

^a^ Note, some individuals appeared on more than one camera.

## 5 DISCUSSION

Spotted hyenas in this rapidly developing landscape appear to be selecting differently for environmental, infrastructural, and human experience characteristics at different scales, demonstrating that it is crucial to consider social–ecological landscape permeability on *coexistence frontiers* and in human‐dominated landscapes generally. We found that crossing parallel wire electric fences may be perceived as risky by hyenas—as indicated by their interactions with the fence largely consisting of either quickly crossing or immediately leaving the area—while simultaneously being highly permeable to this species, which has implications for coexistence and movement for this apex predator. Additionally, the hyenas in this region exhibited several landscape use and navigation propensities that differ from previous studies on this species conducted in landscapes that are on the high or low extremes of anthropogenic influences.

### 5.1 Space use

Hyena ranges were considerably larger than expected given the small size of the two protected areas (Honer et al., 2002; Watts & Holekamp, 2008), potentially further enabled by a relatively high degree of proportional overlap among home ranges. Other studies have shown that adapting to human‐dominated environments may change the fundamental social behaviors of certain carnivore species (e.g., Widdows & Downs, 2015). In our study area, hyenas from different clans exhibited consistent range overlap and are known to frequently enter one another's ranges for anthropogenic resources, such as discarded livestock carcasses (K. Combes, personal observation). This stands in contrast to some other studies (i.e., Barker et al., 2023) and runs counter to the known territorial behaviors of spotted hyenas (Boydston et al., 2001; Watts & Holekamp, 2007), implying potential resource‐ or space‐driven intraspecific social behavior changes which warrant further research.

Hyena home ranges and inter‐clan overlap expanded during the dry season, in contrast to studies in ecologically similar regions that show wildlife tend to disperse more widely in the wet season (Koziarski et al., 2016). Previous research has also shown that spotted hyenas have larger ranges in the wet season due to the seasonal movement and presence of their wild ungulate prey (Trinkel et al., 2004). Similarly, a study on leopards (*Panthera pardus*) in a mixed‐use landscape revealed that they avoided protected areas during the dry season and instead favored tea plantations and forest patches (Naha et al., 2021). Our observed counterintuitive increase in hyena range sizes during the dry season rather than the rainy season could thus be influenced by two factors inherent to this fenced ecosystem. First, due to the electric boundary fences, many ungulate species cannot disperse during the rainy season (Wilkinson, McInturff, et al., 2021), meaning hyenas have little opportunity or need to expand ranges to seasonally track wild prey. Second, the small sizes of the protected areas, coupled with an ongoing rise in the water level of the lake in the national park (James, 2022), may be driving seasonal resource limitations for spotted hyenas and causing them to expand their ranges during the dry season.

### 5.2 Social–ecological landscape navigation and fine‐scale movement decisions

Hyenas in this region selected for different factors at the landscape scale than at a fine scale. Differences in resource selection across scales were particularly apparent for infrastructure and human experience characteristics, and less apparent for environmental characteristics, selection for which largely remained the same across RSF and SSF results. While there were changes in the magnitude of effect, at both broad and fine scales hyenas in this region generally selected for vegetation greenness, lakes, and roads, and against rivers, boundaries, slopes, and high elevation. In arid environments, spotted hyena hydration is mostly derived from prey rather than water sources (Green et al., 1984), and hyenas can also survive for a week or more without water (Holekamp & Dloniak, 2010); thus, selection against rivers at the landscape scale could reflect the aridity of the region. In contrast, lakes featured strongly as important selected habitat for both scales; though the two major lakes in the region are highly alkaline, hyenas have been observed hunting lesser flamingos (*Phoeniconaias minor*), wallowing, and using the lakesides' heavy vegetation as refuge during the heat of the day (authors' observations). Thus, even undrinkable water sources can serve as critical habitat for hyenas (e.g., Matsumoto‐Oda, 2021). The strong selection for greener vegetation at the landscape scale is likely because of the tendency of hyenas to prefer bush and scrub for safety and shade (Kushata et al., 2018; Mwampeta et al., 2021).

Hyena landscape use and navigation appears to be heavily influenced by fine scale interactions with linear infrastructure. Specifically, hyenas moved more quickly and were more likely to have directional persistence when they were farther from boundaries, and they moved more quickly when they were close to roads. Roads have been shown to be important for hyenas to successfully navigate through bushy landscapes (Kushata et al., 2018), despite hyenas' general tendency to avoid human infrastructure (Belton et al., 2016; Green & Holekamp, 2019). Hyenas have been found to move more quickly and in straighter directions when they were near fences (Naha et al., 2023), but the opposite held true for our study. Fence permeability is an important consideration for hyena landscape navigation and populations more broadly (Wilkinson, McInturff, et al., 2021; Williams et al., 2021), yet hyenas and other wildlife may be hesitant to cross fences depending on the animal's perceived risks (i.e., immediate visibility due to microscale habitat) on the opposite side of the fence (Wilkinson, McInturff, et al., 2021). These nuanced scale‐dependent effects likely stem from a combination of factors, with fine‐scale movements dictated by hyena behavioral flexibility, and landscape scale selection dictated by broader resource availability.

At the landscape scale, hyenas are selecting against participatory mapped livestock predation areas and for areas of both verified livestock predation and participatory mapped perceived risk, while fine scale landscape navigation showed no significant selection for these characteristics. The landscape‐scale selection in relation to these human acceptance covariates may indicate that hyenas are broadly selecting for areas in which there may be hostility and mortality risks such as poisoning (i.e., perceived risks from and low tolerance for hyenas), and against areas that people use for livestock grazing (i.e., participatory mapped perceived livestock predation). The latter is supported by previous studies showing that hyenas actively avoid livestock herders (Green & Holekamp, 2019). People are also likely perceiving the highest risks from hyenas in places that constitute suitable hyena habitat, which supports hyena landscape‐scale selection for these areas. In densely populated areas where tolerance is high or there are policies against wildlife killing, carnivore populations may thrive (Athreya et al., 2013; Gebresenbet et al., 2018), yet in areas where tolerance is low (such as within the communities living adjacent to the protected areas in our study site), carnivore populations can be negatively affected by retaliation and other practices (Ripple et al., 2014).

Hyenas in this study site may also be avoiding participatory mapped livestock predation areas—which are likely most often used for grazing—as locations with consistent, predictable risks (i.e., through hostility and higher levels of human activities). This active avoidance of anthropogenic mortality risk has been found in other carnivore species, such as mountain lions (*Puma concolor*) (Smith et al., 2017) and coyotes (*Canis latrans*) (Pershyn et al., 2024), and also reflects findings from previous studies on spotted hyenas in regions with less development, which showed that hyenas reduced their activities or shifted locations in response to human presence (Green & Holekamp, 2019; Kolowski et al., 2007). In contrast to regions of perceived (but unreported) conflict, hyenas selected strongly for regions of verified conflict. This could be due to a combination of factors: verified conflict locations could match optimal combinations of anthropogenic and ecological attractants, as dictated by predation risk mapping theory (e.g., Broekhuis et al., 2017; Miller, 2015), and/or people with higher tolerance and nonlethal deterrents (rather than hostility and lethal deterrents) could be more likely to report their livestock predation instances to the government agency that verifies conflict reports.

#### 5.2.1 Seasonal differences in selection and movement characteristics

Our results also showed seasonal differences in hyena selection for environmental and anthropogenic characteristics. At the landscape scale, in the dry season, hyenas selected for rivers (as opposed to the rainy season when hyenas selected strongly against rivers), selected more strongly against boundaries and areas of perceived livestock predation, and exhibited an increase in selection for roads and participatory mapped risk. The stronger selection for participatory mapped perceived risk during the dry season is likely because people's spatially explicit risk perceptions regarding wildlife have been shown to map onto regions that constitute optimal wildlife habitat or locations where people have frequently encountered particular wildlife species (Read et al., 2021). This region is increasingly prone to intense droughts (Ayugi et al., 2020); future work to support hyena conservation management and conflict prevention in a climate insecure future (Abrahms et al., 2023) could involve working with local community members to better identify and protect micro‐regions of optimal habitat that are simultaneously viewed as risky and not needed or used by communities.

The interactions between movement parameters and linear infrastructure were non‐significant during the dry season, yet in the rainy season hyenas tended to show more consistent directional movement when far from boundaries and to move significantly more quickly when nearer to roads. Given our landscape‐scale results, hyenas are likely behaving differently in relation to roads in the rainy season versus in the dry season. Behavioral states are key determinants of resource selection patterns (Abrahms et al., 2016; Mancinelli et al., 2019). Thus, while some species use roads for easier traversal of vegetatively dense landscapes (Hill et al., 2020), which appears to be the case in our rainy season analysis, hyenas in this study area may also use roads in the dry season for dust bathing, and, within the conservancy, access to intermittently available roadside artificial water points intended for livestock (K. Combes, personal observation). Brown hyenas have also been found to prefer roads in open, less vegetatively dense areas (Welch et al., 2016), possibly for territorial demarcation and ease in locating carrion.

#### 5.2.2 Differences in selection and movement characteristics across land management types

When looking at variation in movement for hyenas living in different management types, at the landscape scale Soysambu hyenas were much more likely to select for roads and against boundaries than were LNNP hyenas. This finding stands in contrast to studies that have found that hyenas living in human‐dominated landscapes actively avoid the more modified parts of those landscapes (Belton et al., 2016) and that hyenas in low disturbance areas demonstrate more risk‐taking behaviors than hyenas in highly disturbed areas (Turner et al., 2019). The constantly high level of anthropogenic activity across all of Soysambu's landscapes (road and otherwise), as well as potential anthropogenic resources along roads (i.e., sporadic artificial water sources), may together overcome or desensitize a Soysambu hyena to the human‐caused “landscape of fear” (Smith et al., 2017; Suraci et al., 2019) within the conservancy. Further, LNNP hyenas selected strongly against roads in their fine scale movements, despite no tourist visitation at night, and heavily regulated vehicle speeds during the day. This implies that LNNP hyenas perceive roads as risky regardless of immediate human activity, which runs counter to previous studies describing hyenas and other species exhibiting fine scale or daily spatiotemporal avoidance of people but otherwise still using infrastructure and other aspects of human‐dominated landscapes (e.g., Boydston et al., 2006; Green & Holekamp, 2019; Toverud, 2019).

The stronger selection of LNNP hyenas for verified livestock predation locations and regions of participatory mapped risk, as well as known fence‐crossing behaviors by LNNP hyenas (Wilkinson, McInturff, et al., 2021), point to a lack of sufficient resources or space in the national park. The water levels in Lake Nakuru have been steadily rising over the past two decades, causing a dramatic expansion of the lake (Muita et al., 2021; Renaut & Owen, 2023) that has greatly reduced the available terrestrial habitat within the enclosed ecosystem. If verified conflict predation locations do indeed reflect combinations of anthropogenic and ecological attractants for hyenas (i.e., Broekhuis et al., 2017), hyenas could increasingly be drawn to these locations outside of the park. Meanwhile, regions of perceived livestock predation were more strongly selected against by LNNP hyenas than by Soysambu hyenas, indicating that hyenas denning in the national park have a stronger aversion to the anthropogenic mortality risks that we have hypothesized are captured by this covariate. This runs counter to previous conclusions about risk‐taking by hyenas in areas of low versus high disturbance (i.e., Turner et al., 2019), but aligns with our results regarding selection for roads by Soysambu hyenas.

### 5.3 Fence behaviors

The abundance of *quick cross* and *bounce* behaviors captured by the barrier behavior analysis, as opposed to walking along the fence or exhibiting average movements near the fence, implies that hyenas may perceive boundaries as risky in this rapidly developing area, and may approach the fence only out of need. When they reach the fence, if they cannot cross, they appear to immediately move away (i.e., *bounce*), and if the fence is permeable, they cross quickly. While McInturff et al. (2020) concluded that fences can create interspecific “ecological winners and losers”, the hyena populations in this region may be a combination of both, depending on the individual, season, land management type, or other factors.

Though our study was able to assess movements of hyenas representing 5 clans, the sample size for assessing fence navigation was limited since not every collared hyena approached the fence lines. Our supplementary camera trap analyses of individual hyenas at the fence line revealed hyenas are approaching the fence and possibly crossing in and out of the national park in extraordinary numbers. Previous studies have suggested that social rank, age, and sex influence spotted hyena risk‐taking behavior (Belton et al., 2018; Green et al., 2018) and space use (Boydston et al., 2003), yet our analysis suggests that individuals spanning different demographics and social ranks may be crossing in and out of the national park. While evidence suggests these behaviors may be underpinned by resource limitations within this relatively small protected area, further research is needed to assess the ecological factors driving these behaviors.

### 5.4 Implications for landscape permeability

Across scales, hyenas in this developing region appear to be selecting for ecological characteristics that largely reflect their resource selection in other, less developed systems (Lala et al., 2022; Mwampeta et al., 2021). However, landscape‐scale navigation also tends to be significantly influenced by covariates describing human perceptions and experiences, while movement choices and behaviors at a finer scale are affected by linear infrastructure. Hyena clan sizes in this region are relatively large (with more than 50 animals per group for clans assessed thus far) despite the small size of the protected areas, which is one potential driver of the apparent movement of hyenas toward people and likely toward anthropogenic resource subsidies, as well as their dry season range expansion. Other studies in similar environments have estimated hyena carrying capacity to be orders of magnitudes lower than the populations seen in our study site (e.g., Holekamp & Smale, 1995; Yirga et al., 2014). Within *coexistence frontiers*, resource constraints and anthropogenic activities may create contexts in which anthropogenic factors are highly reliable predictors of hyena movement and population dynamics, despite this species' role as a notable generalist (Holekamp & Dloniak, 2010; Yirga et al., 2017).

Despite this suspected significant reliance on anthropogenic resources, hyenas showed different preferences and selection strengths for and against roads depending on scale and land management type. This variation is contrary to what we expected, but indicates the importance of considering context (i.e., behavioral state and associated needs)—as supported by numerous road ecology studies (see Hill et al., 2020)—when considering the role of roads in landscape connectivity. Fences also present a semi‐permeable barrier for spotted hyenas, which appear to cross them as quickly as possible rather than lingering, and largely avoid them at the landscape scale. Other studies have found that keeping development and subdivision below a certain threshold may allow for sustained carnivore navigation of the landscape between core habitat areas (Smith et al., 2019; Xu et al., 2023). This may also prove true for the spotted hyenas, which appear to have complex relationships with infrastructure within and surrounding the two protected areas. Yet, hyena relationships with fences can also provide information that is helpful for management efforts. We can use fence behavior analyses to determine existing permeable fence segments (Xu et al., 2020) and make ecologically‐informed decisions about where carnivore (and other wildlife) corridors in and out of fenced regions will be the most useful, practical, and cost‐effective.

Overall, our results imply that anthropogenic factors may influence fine scale decision making differently than landscape‐scale selection. Hyenas may be adaptable enough to switch to anthropogenic food sources in regions of depleted natural prey or limited resources, yet their ability to rely on anthropogenic food may be linked to regional tolerance of hyenas (e.g., Yirga et al., 2013). Understanding in which contexts regions of spatially explicit human acceptance and experience are more or less likely to be selected by wildlife can have the potential to predict where wildlife corridors are likely to succeed for certain species or taxa, while also providing insight into how wildlife may be using anthropogenic resources (Behr et al., 2017; Ghoddousi et al., 2021). Coupled with hyena context‐specific selection for and against infrastructure characteristics, these results demonstrate that a multiscale and multidisciplinary understanding of social–ecological landscape use and navigation can help to determine where and when this species may thrive in human‐dominated landscapes. This approach is essential for a species that is key for removing carcasses and diseases from the environment (Sonawane et al., 2021), and in a location that is becoming increasingly fenced, but the social–ecological approaches used here can also be applied to movements and reintroductions of other controversial wildlife species in other settings (see Ditmer et al., 2022; Manfredo et al., 2021; Vasudev et al., 2023; Williamson et al., 2023; Williamson & Sage, 2020). Future research on social–ecological landscape permeability for wildlife should include the incorporation of detailed land cover covariates, in‐depth quantification of tolerance and experience as spatial proxies for risks and benefits that underpin animal behaviors, and testing of GPS collar data across RSF‐ and SSF‐informed social–ecological least cost corridor models.

## 6 CONCLUSION

Spotted hyenas are one of the most behaviorally flexible large carnivore species. Yet, their reputation for adaptiveness has previously discouraged studies on whether and to what extent people impact their movements and behaviors. As a widespread species across Africa, spotted hyenas provide us with a litmus test for understanding carnivore abilities to live alongside people and navigate landscapes on coexistence frontiers. Yet, we also know that coexistence requires adaptation by both people and carnivores to succeed (Chapron et al., 2014). This study has demonstrated that integrating spatial and contextual information on ecology, infrastructure, and human tolerance and experiences can help us to better examine how carnivores may adjust to proliferating human disturbances and navigate human‐dominated landscapes at different scales. By gaining these holistic understandings of the effects of ongoing global urbanization on behaviorally flexible, ecologically critical species, we may be able to design and redesign anthropogenic landscapes that prioritize both ecological resilience and environmental justice.

## AUTHOR CONTRIBUTIONS

**Christine E. Wilkinson:** Conceptualization (lead); data curation (lead); formal analysis (lead); funding acquisition (lead); investigation (lead); methodology (lead); project administration (lead); resources (lead); visualization (lead); writing – original draft (lead); writing – review and editing (lead). **Wenjing Xu:** Formal analysis (equal); methodology (supporting); writing – original draft (supporting); writing – review and editing (equal). **Amalie Luneng Solli:** Data curation (equal); formal analysis (supporting); visualization (supporting); writing – review and editing (supporting). **Justin S. Brashares:** Conceptualization (supporting); supervision (equal); validation (supporting); writing – review and editing (equal). **Christine Chepkisich:** Conceptualization (supporting); investigation (supporting); resources (supporting); writing – review and editing (supporting). **Gerald Osuka:** Investigation (supporting); writing – review and editing (supporting). **Maggi Kelly:** Conceptualization (supporting); funding acquisition (supporting); supervision (equal); validation (equal); writing – review and editing (equal).

## CONFLICT OF INTEREST STATEMENT

The authors declare no conflict of interest.

## Supporting information


Appendix S1

## Data Availability

Data are currently archived in a public repository (Dryad) for peer review (https://datadryad.org/stash/share/jw1Nol_UG‐rpGQMHE9f49gkh9BeYVcsAfoubF‐N77sg), and will be made public upon acceptance of this manuscript (https://doi.org/10.5061/dryad.0vtb8h52).
