# Supplementary material for: Social–ecological predictors of spotted hyena navigation through a shared landscape
Source: Ecol Evol. 2024 Apr 25;14(4):e11293. doi: 10.1002/ece3.11293 (PMC11045923; doi:10.1002/ece3.11293)
Supplement: Supplementary file 1 — Appendix S1 [file ECE3-14-e11293-s001.docx]

**Supporting Information**


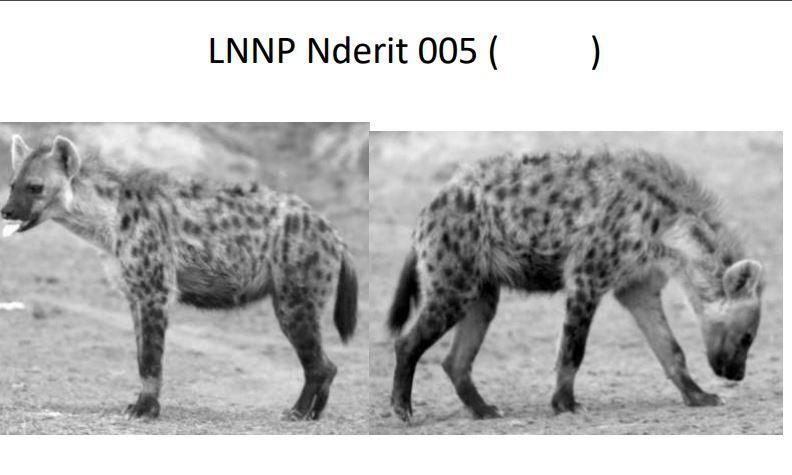


Supplementary Figure 1. Example of left and right side photographs used to identify individual spotted hyenas.


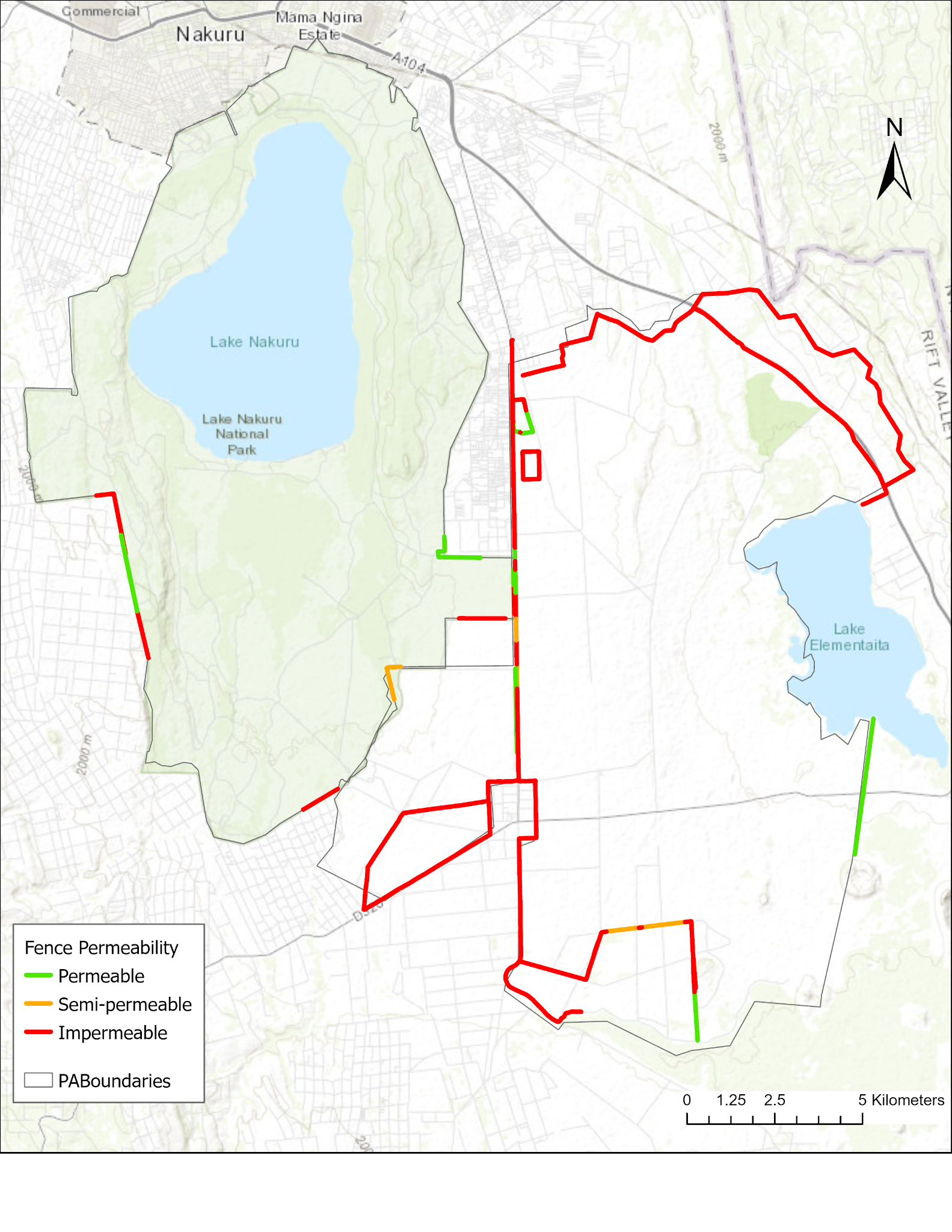


Supplementary Figure 2. Permeable, semi-permeable, and impermeable portions of the protected area fences as revealed by Barrier Behaviour Analysis results. Permeability of other stretches of the boundaries is unknown due to lack of sufficient hyena encounters.


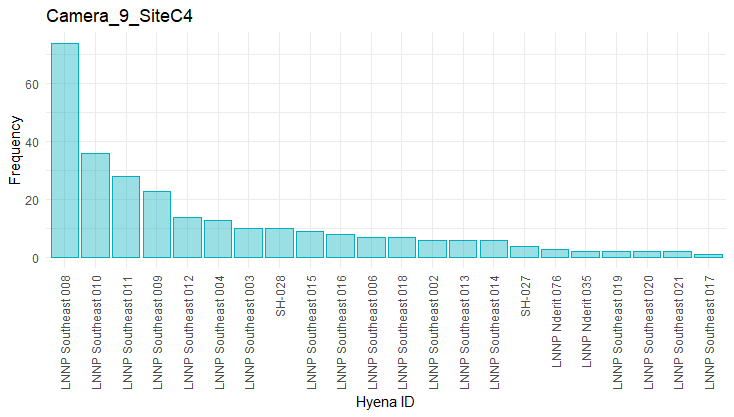


Supplementary Figure 3. An example showing photo frequency for individual spotted hyenas appearing at the fence at one camera site (Site C4).

Supplementary Table 1. Coefficients and deviance for global resource selection models using varying ratios of available to used points.

|  | **1:1 ratio** | **2:1 ratio** | **3:1 ratio** | **4:1 ratio** |
| --- | --- | --- | --- | --- |
| NDVI | 0.053501 | 0.062996 | 0.051813 | 0.04429 |
| Elevation | -0.0674 | -0.05897 | -0.06983 | -0.05496 |
| Slope | -0.02917 | -0.03065 | -0.02461 | -0.02186 |
| Distance to rivers | 0.05183 | 0.050252 | 0.051741 | 0.039985 |
| Distance to roads | -0.08378 | -0.07947 | -0.07377 | -0.07858 |
| Distance to lakes | -0.13311 | -0.12399 | -0.12728 | -0.13559 |
| Distance to boundaries | 0.161996 | 0.159437 | 0.159024 | 0.166902 |
| Distance to perceived risk | -0.27254 | -0.26433 | -0.26563 | -0.2635 |
| Distance to perceived conflict | 0.221286 | 0.21869 | 0.217945 | 0.219951 |
| Distance to verified conflict | -0.13274 | -0.13767 | -0.14731 | -0.14013 |
| Deviance | 44444.58 | 61302.69 | 72242.56 | 80501.17 |

Supplementary Table 2. Proportion of home range overlap (95% KDE) between individual pairs of spotted hyenas overall, during the rainy season, and during the dry season. Directionality is via horizontal rows (i.e., each row indicates the proportion of that hyena’s home range overlap with the home range of another hyena).

| *Data subset* | *ID* | *1623* | *1624* | *1626* | *1627* | *1628* | *1630* |
| --- | --- | --- | --- | --- | --- | --- | --- |
| All data | **1623** | 1 | 0.278 | 0 | 0.09 | 0.532 | 0 |
|  | **1624** | 0.159 | 1 | 0.047 | 0.074 | 0.095 | 0.038 |
|  | **1626** | 0 | 0.136 | 1 | 0 | 0 | 0.941 |
|  | **1627** | 0.115 | 0.167 | 0 | 1 | 0.069 | 0 |
|  | **1628** | 0.167 | 0.052 | 0 | 0.017 | 1 | 0 |
|  | **1630** | 0 | 0.103 | 0.881 | 0 | 0 | 1 |
| Rainy season | **1623** | 1 | 0.289 | 0 | 0.09 | 0.318 | 0 |
|  | **1624** | 0.149 | 1 | 0.032 | 0.056 | 0.006 | 0.029 |
|  | **1626** | 0 | 0.112 | 1 | 0 | 0 | 0.946 |
|  | **1627** | 0.124 | 0.15 | 0 | 1 | 0 | 0 |
|  | **1628** | 0.123 | 0.005 | 0 | 0 | 1 | 0 |
|  | **1630** | 0 | 0.087 | 0.825 | 0 | 0 | 1 |
| Dry season | **1623** | 1 | 0.282 | 0 | 0.104 | 0.702 | 0 |
|  | **1624** | 0.183 | 1 | 0.071 | 0.065 | 0.156 | 0.057 |
|  | **1626** | 0 | 0.17 | 1 | 0 | 0 | 0.931 |
|  | **1627** | 0.128 | 0.123 | 0 | 1 | 0.21 | 0 |
|  | **1628** | 0.202 | 0.069 | 0 | 0.049 | 1 | 0 |
|  | **1630** | 0 | 0.135 | 0.921 | 0 | 0 | 1 |
|  | | | | | | | |
| *Inter-clan summary* | *mean* | *Std.dev* | *maximum* |  |  |  |  |
| All data | 0.076 | 0.115 | 0.278 |  |  |  |  |
| Rainy season | 0.056 | 0.087 | 0.318 |  |  |  |  |
| Dry season | 0.097 | 0.144 | 0.702 |  |  |  |  |

Supplementary Table 3. Bhattacharya coefficient values for home range overlap (95% KDE) between individual pairs of spotted hyenas overall, during the rainy season, and during the dry season.

| *Data subset* | *ID* | *1623* | *1624* | *1626* | *1627* | *1628* | *1630* |
| --- | --- | --- | --- | --- | --- | --- | --- |
| All data | 1623 | 1 | 0.159 | 0 | 0.061 | 0.2 | 0 |
| All data | 1624 | 0.159 | 1 | 0.036 | 0.039 | 0.028 | 0.027 |
| All data | 1626 | 0 | 0.036 | 1 | 0 | 0 | 0.956 |
| All data | 1627 | 0.061 | 0.039 | 0 | 1 | 0.013 | 0 |
| All data | 1628 | 0.2 | 0.028 | 0 | 0.013 | 1 | 0 |
| All data | 1630 | 0 | 0.027 | 0.956 | 0 | 0 | 1 |
| Dry season | 1623 | 1 | 0.178 | 0 | 0.0659 | 0.258 | 0 |
| Dry season | 1624 | 0.178 | 1 | 0.05 | 0.037 | 0.041 | 0.046 |
| Dry season | 1626 | 0 | 0.05 | 1 | 0 | 0 | 0.958 |
| Dry season | 1627 | 0.0659 | 0.037 | 0 | 1 | 0.062 | 0 |
| Dry season | 1628 | 0.258 | 0.041 | 0 | 0.062 | 1 | 0 |
| Dry season | 1630 | 0 | 0.046 | 0.958 | 0 | 0 | 1 |
| Rainy season | 1623 | 1 | 0.157 | 0 | 0.06 | 0.15 | 0 |
| Rainy season | 1624 | 0.157 | 1 | 0.023 | 0.035 | 0.003 | 0.017 |
| Rainy season | 1626 | 0 | 0.023 | 1 | 0 | 0 | 0.93 |
| Rainy season | 1627 | 0.06 | 0.035 | 0 | 1 | 0 | 0 |
| Rainy season | 1628 | 0.15 | 0.003 | 0 | 0 | 1 | 0 |
| Rainy season | 1630 | 0 | 0.017 | 0.93 | 0 | 0 | 1 |
|  | | | | | | | |
| *Inter-clan summary* | *Mean* | *Std.dev* | *Maximum* |  |  |  |  |
| All data | 0.04 | 0.063 | 0.159 |  |  |  |  |
| Dry season | 0.053 | 0.076 | 0.178 |  |  |  |  |
| Rainy season | 0.032 | 0.055 | 0.157 |  |  |  |  |

Supplementary Table 4. Relative importance of variables for resource selection functions and step selection functions.

| *Variable* | *RSF* | *SSF* |
| --- | --- | --- |
| NDVI | 0.095 | 0.078 |
| Elevation | 0.056 | 0.108 |
| Slope | 0.008 | 0.022 |
| Distance to rivers | 0.05 | 0.085 |
| Distance to lakes | 0.123 | 0.185 |
| Distance to roads | 0.066 | 0.137 |
| Distance to boundaries | 0.1 | 0.195 |
| Distance to perceived livestock predation locations | 0.191 | 0.058 |
| Distance to verified livestock predation locations | 0.142 | 0.13 |
| Distance to locations perceived as risky due to hyenas | 0.157 | 0.002 |

Supplementary Table 5. Seasonal step selection of environmental and infrastructure covariates for a hyena that frequently travels between Lake Nakuru National Park and Soysambu Conservancy, Kenya.

| **Dry season** | | | | | | |
| --- | --- | --- | --- | --- | --- | --- |
| *Variable* | *coeff* | *se* | *z-value* | | *p-value* | |
| NDVI | -0.134 | 0.022 | -9.366 | | <0.001 | |
| Elevation | -0.257 | 0.072 | -4.338 | | <0.001 | |
| Slope | 0.06 | 0.026 | 6.72 | | <0.001 | |
| Distance to rivers | 0.365 | 0.15 | 1.912 | | 0.056 | |
| Distance to roads | 1.014 | 0.052 | 9.053 | | <0.001 | |
| Distance to lakes | -0.003 | 0.068 | -0.053 | | 0.958 | |
| Distance to boundaries | 0.117 | 0.068 | 1.453 | | 0.146 | |
| Distance to perceived livestock predation locations | 0.091 | 0.089 | 1.03 | | 0.303 | |
| Distance to verified livestock predation locations | -0.404 | 0.092 | -12.772 | | <0.001 | |
| Distance to locations perceived as risky due to hyenas | 0.293 | 0.065 | 5.668 | | <0.001 | |
| Distance to boundaries:log_sl | -0.014 | 0.005 | -1.267 | | | 0.205 |
| Distance to boundaries:cos_ta | 0.035 | 0.14 | 3.419 | | | <0.001 |
| Distance to roads:log_sl | -0.142 | 0.008 | -9.238 | | | <0.001 |
|  | | | | | | |
| **Rainy season** | | | | | | |
| *Variable* | *coeff* | *se* | | *z-value* | | *p-value* |
| NDVI | 0.078 | 0.032 | | 0.96 | | 0.337 |
| Elevation | 0.00003 | 0.109 | | 0 | | 0.9997 |
| Slope | -0.061 | 0.038 | | -2.119 | | 0.034 |
| Distance to rivers | 0.428 | 0.19 | | 1.836 | | 0.066 |
| Distance to roads | 0.89 | 0.062 | | 4.973 | | <0.001 |
| Distance to lakes | 0.014 | 0.089 | | 0.134 | | 0.893 |
| Distance to boundaries | -0.265 | 0.086 | | -1.134 | | 0.255 |
| Distance to perceived livestock predation locations | 0.348 | 0.116 | | 4.34 | | <0.001 |
| Distance to verified livestock predation locations | -0.593 | 0.114 | | -6.852 | | <0.001 |
| Distance to locations perceived as risky due to hyenas | 0.457 | 0.085 | | 4.872 | | <0.001 |
| Distance to boundaries:log_sl | 0.01 | 0.006 | | 0.402 | | 0.688 |
| Distance to boundaries:cos_ta | 0.083 | 0.017 | | 1.621 | | 0.105 |
| Distance to roads:log_sl | -0.11 | 0.009 | | -3.932 | | <0.001 |

Supplementary Table 6. Frequencies of individual hyena appearances at fence cameras.

| **Hyena ID** | **Frequency** |
| --- | --- |
| LNNP Naishi 011 | 26 |
| LNNP Naishi 012 | 10 |
| LNNP Naishi 013 | 17 |
| LNNP Naishi 014 | 2 |
| LNNP Naishi 015 | 20 |
| LNNP Naishi 016 | 15 |
| LNNP Naishi 018 | 1 |
| LNNP Naishi 019 | 3 |
| LNNP Naishi 020 | 2 |
| LNNP Naishi 021 | 33 |
| LNNP Naishi 022 | 1 |
| LNNP Naishi 023 | 2 |
| LNNP Naishi 024 | 4 |
| LNNP Naishi 025 | 1 |
| LNNP Naishi 026 | 1 |
| LNNP Naishi 027 | 8 |
| LNNP Naishi 029 | 3 |
| 0.00.LNNP Naishi 030 | 4 |
| LNNP Naishi 031 | 2 |
| LNNP Naishi 032 | 3 |
| LNNP Naishi 033 | 3 |
| LNNP Naishi 034 | 7 |
| LNNP Naishi 035 | 4 |
| LNNP Naishi 036 | 45 |
| LNNP Naishi 037 | 3 |
| LNNP Naishi 038 | 8 |
| LNNP Naishi 039 | 4 |
| LNNP Naishi 040 | 8 |
| LNNP Naishi 041 | 12 |
| LNNP Naishi 042 | 2 |
| LNNP Naishi 044 | 2 |
| LNNP Naishi 045 | 2 |
| LNNP Naishi 046 | 15 |
| LNNP Naishi 047 | 4 |
| LNNP Naishi 048 | 2 |
| LNNP Naishi 049 | 4 |
| LNNP Naishi 050 | 1 |
| LNNP Naishi 051 | 2 |
| LNNP Naishi 052 | 2 |
| LNNP Naishi 053 | 7 |
| LNNP Naishi 054 | 4 |
| LNNP Naishi 055 | 18 |
| LNNP Naishi 056 | 9 |
| LNNP Naishi 057 | 5 |
| LNNP Naishi 058 | 13 |
| LNNP Naishi 059 | 2 |
| LNNP Naishi 060 | 2 |
| LNNP Naishi 061 | 2 |
| LNNP Naishi 062 | 2 |
| LNNP Naishi 063 | 2 |
| LNNP Naishi 064 | 8 |
| LNNP Naishi 065 | 2 |
| LNNP Naishi 066 | 2 |
| LNNP Naishi 067 | 4 |
| LNNP Naishi 068 | 6 |
| LNNP Naishi 069 | 6 |
| LNNP Naishi 070 | 2 |
| LNNP Naishi 071 | 2 |
| LNNP Naishi 072 | 2 |
| LNNP Naishi 073 | 2 |
| LNNP Naishi 074 | 2 |
| LNNP Naishi 075 | 4 |
| LNNP Naishi 076 | 2 |
| LNNP Nderit 001 | 8 |
| LNNP Nderit 002 | 1 |
| LNNP Nderit 003 | 20 |
| LNNP Nderit 005 | 232 |
| LNNP Nderit 006 | 36 |
| LNNP Nderit 009 | 113 |
| LNNP Nderit 010 | 9 |
| LNNP Nderit 011 | 53 |
| LNNP Nderit 014 | 3 |
| LNNP Nderit 016 | 289 |
| LNNP Nderit 017 | 18 |
| LNNP Nderit 018 | 2 |
| LNNP Nderit 020 | 229 |
| LNNP Nderit 021 | 22 |
| LNNP Nderit 022 | 214 |
| LNNP Nderit 023 | 44 |
| LNNP Nderit 024 | 4 |
| LNNP Nderit 029 | 105 |
| LNNP Nderit 030 | 13 |
| LNNP Nderit 031 | 18 |
| LNNP Nderit 035 | 160 |
| LNNP Nderit 036 | 64 |
| LNNP Nderit 037 | 8 |
| LNNP Nderit 038 | 1 |
| LNNP Nderit 039 | 2 |
| LNNP Nderit 040 | 40 |
| LNNP Nderit 041 | 3 |
| LNNP Nderit 042 | 14 |
| LNNP Nderit 043 | 17 |
| LNNP Nderit 044 | 27 |
| LNNP Nderit 045 | 12 |
| LNNP Nderit 046 | 12 |
| LNNP Nderit 047 | 57 |
| LNNP Nderit 048 | 2 |
| LNNP Nderit 049 | 272 |
| LNNP Nderit 050 | 24 |
| LNNP Nderit 051 | 12 |
| LNNP Nderit 054 | 6 |
| LNNP Nderit 055 | 5 |
| LNNP Nderit 056 | 2 |
| LNNP Nderit 058 | 7 |
| LNNP Nderit 059 | 143 |
| LNNP Nderit 060 | 3 |
| LNNP Nderit 061 | 10 |
| LNNP Nderit 062 | 18 |
| LNNP Nderit 063 | 1 |
| LNNP Nderit 064 | 14 |
| LNNP Nderit 065 | 2 |
| LNNP Nderit 066 | 2 |
| LNNP Nderit 067 | 2 |
| LNNP Nderit 068 | 2 |
| LNNP Nderit 069 | 13 |
| LNNP Nderit 071 | 17 |
| LNNP Nderit 072 | 8 |
| LNNP Nderit 073 | 13 |
| LNNP Nderit 074 | 26 |
| LNNP Nderit 075 | 26 |
| LNNP Nderit 076 | 34 |
| LNNP Nderit 077 | 20 |
| LNNP Nderit 078 | 4 |
| LNNP Nderit 079 | 2 |
| LNNP Nderit 080 | 2 |
| LNNP Nderit 081 | 2 |
| LNNP Nderit 082 | 4 |
| LNNP Nderit 083 | 83 |
| LNNP Nderit 084 | 9 |
| LNNP Nderit 086 | 16 |
| LNNP Nderit 087 | 6 |
| LNNP Nderit 088 | 13 |
| LNNP Nderit 089 | 63 |
| LNNP Nderit 090 | 2 |
| LNNP Nderit 091 | 4 |
| LNNP Nderit 092 | 8 |
| LNNP Southeast 001 | 7 |
| LNNP Southeast 002 | 8 |
| LNNP Southeast 003 | 14 |
| LNNP Southeast 004 | 21 |
| LNNP Southeast 005 | 11 |
| LNNP Southeast 006 | 9 |
| LNNP Southeast 007 | 8 |
| LNNP Southeast 008 | 112 |
| LNNP Southeast 009 | 95 |
| LNNP Southeast 010 | 90 |
| LNNP Southeast 011 | 82 |
| LNNP Southeast 012 | 18 |
| LNNP Southeast 013 | 47 |
| LNNP Southeast 014 | 20 |
| LNNP Southeast 015 | 59 |
| LNNP Southeast 016 | 14 |
| LNNP Southeast 017 | 1 |
| LNNP Southeast 018 | 19 |
| LNNP Southeast 019 | 2 |
| LNNP Southeast 020 | 18 |
| LNNP Southeast 021 | 2 |
| LNNP Southeast 022 | 2 |
| LNNP Southeast 023 | 13 |
| LNNP Southeast 024 | 8 |
| LNNP Southeast 025 | 2 |
| LNNP Southeast 026 | 20 |
| LNNP Southeast 027 | 6 |
| LNNP Southeast 028 | 4 |
| LNNP Southeast 029 | 2 |
| LNNP Southeast 030 | 2 |
| LNNP Southeast 031 | 10 |
| LNNP Southeast 032 | 2 |
| LNNP Southeast 033 | 4 |
| LNNP Southeast 034 | 2 |
| LNNP Southeast 036 | 8 |
| LNNP Southeast 037 | 2 |
| LNNP Southeast 038 | 2 |
| LNNP Southeast 039 | 4 |
| LNNP Southeast 040 | 8 |
| LNNP Southeast 041 | 6 |
| LNNP Southeast 042 | 2 |
| LNNP Southeast 043 | 7 |
| LNNP Southeast 044 | 4 |
| LNNP Southeast 045 | 21 |
| LNNP Southeast 046 | 6 |
| LNNP Southeast 047 | 6 |
| LNNP Southeast 048 | 1 |
| LNNP Southeast 049 | 4 |
| LNNP Southeast 050 | 4 |
| LNNP Southeast 051 | 2 |
| LNNP Southeast 052 | 2 |
| LNNP Southeast 053 | 2 |
| LNNP Southeast 054 | 2 |
| LNNP Southeast 055 | 2 |
| LNNP Southeast 056 | 4 |
| LNNP Southeast 057 | 1 |
| LNNP Southeast 058 | 5 |
| LNNP Southeast 059 | 2 |
| LNNP Southeast 060 | 2 |
| LNNP Southeast 061 | 2 |
| LNNP Southeast 062 | 2 |
| LNNP Southeast 063 | 2 |
| LNNP Southeast 064 | 2 |
| LNNP Southeast 065 | 2 |
| LNNP Southeast 066 | 4 |
| LNNP Southeast 067 | 2 |
| LNNP Southeast 068 | 8 |
| LNNP Southeast 069 | 6 |
| LNNP Southeast 070 | 2 |
| LNNP Southeast 071 | 9 |
| LNNP Southeast 072 | 2 |
| LNNP Southeast 073 | 10 |
| LNNP Southeast 074 | 1 |
| LNNP Southeast 075 | 10 |
| LNNP Southeast 076 | 6 |
| LNNP Southeast 077 | 4 |
| LNNP Southeast 078 | 2 |
| LNNP Southeast 079 | 6 |
| LNNP Southeast 080 | 2 |
| LNNP Southeast 081 | 4 |
| LNNP Southeast 082 | 2 |
| LNNP Southeast 083 | 1 |
| LNNP Southeast 084 | 2 |
| LNNP Southeast 085 | 2 |
| LNNP Southeast 086 | 1 |
| SH-027 | 90 |
| SH-028 | 60 |
| SH-096 | 1723 |
| SH-097 | 4 |

Autocorrelation function. We used the autocorrelation function below (using R) to assess and plot the autocorrelation in our data prior to destructively sampling the data and initiating step selection functions.

acf.test <- function (residuals, id, type = c("correlation", "covariance","partial"), ci = 0.95)

{

type <- match.arg(type)

acfk <- lapply(levels(factor(id)), function(x) acf(residuals[id == x], type = type, plot = FALSE))

threshold <- unlist(lapply(acfk, function(x) qnorm((1 + ci)/2)/sqrt(x$n.used)))

lag <- unlist(lapply(1:length(acfk), function(i) which(acfk[[i]]$acf < threshold[i])[1]))

return(list(acfk = acfk, threshold = threshold, lag = lag))

}
